# Inferring fitness seascapes from evolutionary histories

**DOI:** 10.1101/2025.06.08.658500

**Published:** 2025-06-08

**Authors:** Yirui Gao, Brian Lee, John P. Barton

**Affiliations:** 1 Department of Physics and Astronomy, University of California, Riverside, USA.; 2 Department of Computational and Systems Biology, University of Pittsburgh School of Medicine, USA.; 3 Department of Physics and Astronomy, University of Pittsburgh, USA.

## Abstract

Evolutionary dynamics are often conceptualized as walks on an adaptive landscape, with populations climbing toward optimal fitness peaks. However, environmental changes can transform static landscapes into dynamic “fitness seascapes” where natural selection fluctuates in time. Here, we used a path integral approach derived from statistical physics to reveal time-varying selection pressures from genetic sequence data. We found that constraints on the fitness seascape are determined by the screened Poisson equation, which also describes the screening of electric fields in media with mobile charge carriers. In our model, changes in mutation frequencies act as “charges” that reveal the underlying fitness seascape, which is analogous to an electrostatic potential. After validating our method in simulations, we applied it to study how HIV-1 evolves to escape immune control by T cells within individual hosts. Our analysis showed that the fitness benefit of immune escape declines as T cell responses approach their peak intensity, suggesting that functional exhaustion may impair the effectiveness of the immune response against HIV-1. Overall, our approach provides a general framework for capturing the complex dynamics of natural selection in rapidly evolving populations.

## Introduction

The concept of natural selection is central to evolution. Reproductively successful individuals pass on their genes to subsequent generations, driving genetic change through the “survival of the fittest.” This process is often visualized through fitness landscapes, where each point represents a possible genotype and its elevation corresponds to reproductive fitness. In this metaphor, evolution can be seen as populations climbing uphill toward fitness peaks while avoiding low-fitness valleys.

However, the fitness effects of traits or mutations can change along with the environment. In one of the most famous examples of natural selection, darkly colored moths became favored over lightly pigmented variants during the Industrial Revolution, as their colors provided better camouflage in smoky environments^1,2^. When pollution levels declined, lightly colored moths returned. Modern analyses have also highlighted fluctuating selection in other natural populations^3–8^. This dynamic is captured by the concept of fitness seascapes, where the landscape itself shifts over time^9^.

Temporal genetic data – sequences sampled over time from sources such as experimental evolution, genomic surveillance of pathogens, or ancient DNA^10–13^ – provide valuable windows into population dynamics that can be leveraged to understand evolution. These data capture changes in the genetic structure of populations as they occur in response to shifting fitness constraints. However, signals of adaptation must be carefully separated from other stochastic contributions to population dynamics.

At present, several methods have been developed to learn fluctuating fitness effects from data^8,14–16^. One limitation of current approaches is that they focus on the fitness effects of single mutations, without explicitly considering the effects of the genetic background. This simplification risks over-looking important contributions to evolution. For example, beneficial mutations can be driven to extinction through competition with even fitter variants in a process known as clonal interference^17^. Conversely, neutral mutations can hitchhike to high frequencies when they appear on high-fitness genetic backgrounds^18^. These complex interactions highlight the need for approaches that account for the genetic context in which evolution occurs. Alternatively, a parallel line of work has studied how fitness (and changes in fitness) is reflected in evolutionary dynamics over time, but without estimating the individual contributions of specific mutations^19,20^.

Here, we developed a solution to this problem using methods from statistical physics. Starting from a stochastic model of population evolution, we derived a path integral that quantifies the relative likelihood of different evolutionary histories. One can then obtain an analytical equation for the time-varying fitness effects of mutations that best explain the observed trajectory of evolution. Remarkably, this expression maps onto the screened Poisson equation from physics. In our inference framework, time plays the role of a spatial coordinate, and the fitness effects of mutations are like an electric potential. Changes in the frequencies of mutations act like charges that describe the shape of the fitness seascape at a particular moment in time. We validate our approach in simulations and apply it to study how human immunodeficiency virus (HIV)-1 evolves within individual hosts.

## Results

### Evolutionary model

Our starting point for analysis is the classic Wright-Fisher (WF) model^21^, which describes the dynamics of a population of N individuals subject to mutations, genetic recombination, and natural selection. For simplicity, we will represent the haploid genetic sequence of each individual a as a binary string $g^{a}=\left(g_{1}^{a},g_{2}^{a},\ldots ,g_{\ell }^{a}\right)$ with $g_{i}^{a}\in \{0,1\}$. Here the index i labels each site in the genetic sequence, running from 1 to ℓ, and a labels the genotype (of which there are $M=2^{\ell }$ possibilities). The probability of mutation per site per reproduction cycle is μ, which we fix in time. Mutation at a site i converts the value of the genetic sequence at that site, $g_{i}$, from the wild-type (WT, represented by 0) to the mutant (1) state, or vice versa.

Recombination refers to the process of genetic exchange between individuals, producing a “child” genome with shuffled contributions from each of the “parents.” While this phenomenon is most familiar in sexual reproduction, it can also occur in other contexts, including HIV-1 replication within host cells^22^. We write the probability of a recombination breakpoint occurring between any adjacent sites in the genome as r, which we also assume to be time-independent. When recombination occurs between two parents a and b at site i, the resulting offspring c inherits positions 1 through i from parent a and i+1 through ℓ from parent b, creating the sequence $g^{c}=\left(g_{1}^{a},g_{2}^{a},\ldots ,g_{i}^{a},g_{i+1}^{b},\ldots ,g_{\ell }^{b}\right)$.

To model natural selection, we assign a time-dependent fitness value $f_{a}(t)$ to each genotype a. This fitness value directly influences reproductive success: genotypes with higher fitness values contribute more offspring to the next generation. We can express the fitness function as a polynomial expansion of genetic contributions,

$$f_{a}\left(t\right)=1+\sum_{i=1}^{\ell }s_{i}\left(t\right)g_{i}^{a}+\sum_{i=1}^{\ell }\sum_{j>i}s_{ij}\left(t\right)g_{i}^{a}g_{j}^{a}+\ldots ,$$

where the first-order terms $s_{i}(t)$ represent the direct fitness effects of individual mutations and higher-order terms capture interactions between mutations. While this framework can accommodate arbitrarily complex fitness landscapes with many interaction terms, we focus on the simpler case where only first-order terms $s_{i}(t)$ are included. This approximation captures the essential dynamics of a shifting fitness seascape while remaining mathematically tractable. In population genetics, the $s_{i}(t)$ are called selection coefficients and measure the fitness advantage or disadvantage of each mutation.

We define $n_{a}(t)$ as the number of individuals with genotype a at time t, and $z_{a}(t)=n_{a}(t)/N$ as the corresponding frequency. The state of the population is described by a genotype frequency vector $z(t)=\left(z_{1}(t),z_{2}(t),\ldots ,z_{M}(t)\right)$. In the WF model, evolution proceeds stochastically. The state of the population in the next generation, z(t+1), follows a multinomial distribution

(1) $$P\left(z\left(t+1\right)|z\left(t\right)\right)=N!\prod_{a=1}^{M}\frac{{\left(p_{a}\left(t\right)\right)}^{Nz_{a}\left(t+1\right)}}{\left(Nz_{a}\left(t+1\right)\right)!}.$$

Here, $p_{a}(t)$ represents the expected frequency of genotype a in the next generation, incorporating the effects of natural selection, mutation, and recombination (with detailed expressions presented in Supplementary Information).

### Inference of fitness seascapes

In principle, we could use the transition probability in equation (1) to estimate selection coefficients from observed evolutionary trajectories. Following principles of Bayesian inference^23^, the posterior probability of the selection coefficients $s(t)=\left(s_{1}(t),s_{2}(t),\ldots ,s_{\ell }(t)\right)$ from one generation of evolution is

$$P_{\text{post}}(s(t))=P(z(t+1)|z(t),s(t))\times P_{\text{prior}}(s(t)).$$

The first term represents how likely we are to observe the actual evolutionary trajectory given particular selection coefficients, and $P_{\text{prior}}(s(t))$ encodes our prior knowledge about plausible selection coefficient values. However, the functional form of (1) is complicated, making this expression difficult to work with directly.

To make this problem tractable, we apply the diffusion approximation of the WF model^21,24,25^. This mathematical approach considers the scaling limit in which the population size N→∞, while the selection coefficients, mutation rate, and recombination rate are all 𝒪(1/N) (Supplementary Information; see also ref.^26^. In this limit, we obtain a continuous-time stochastic model of population evolution. The probability of an evolutionary trajectory, or “path,” of genotype frequencies from times $t_{0}$ to $t_{K}$, conditioned on the initial state of the population $z\left(t_{0}\right)$, can then be expressed as a path integral^26^ (see also refs.^27,28^)

$$ℒ\left((z(t){)}_{t=t_{0}}^{t_{K}}\right)\propto \text{exp}\left[-\frac{N}{2}S\left((z(t){)}_{t=t_{0}}^{t_{K}}\right)\right],$$

with

$$S\left((z(t){)}_{t=t_{0}}^{t_{K}}\right)=\int_{t_{0}}^{t_{K}}dt\;L(z(t))\;=\int_{t_{0}}^{t_{K}}dt\left[\left(\dot{z}-d(z){)}^{T}C(z{)}^{-1}(\dot{z}-d(z)\right)\right].$$

Here and below, we suppress the time indices on the genotype frequency vectors z(t) and other time-varying quantities when their time dependence is clear. In the above expression, d(z) is the drift vector, representing the deterministic forces of selection, mutation, and recombination driving evolution, and C(z) is the diffusion matrix, which captures stochastic fluctuations (Supplementary Information).

Imposing additional constraints on the s(t) through the prior distribution $P_{\text{prior}}(s(t))$ can improve our ability to estimate realistic selection coefficients. We assume that most mutations have little effect on fitness, which we quantify through a Gaussian distribution with mean zero and variance 1/Nγ for the selection coefficients,

$$P_{\text{prior}}\left((s(t){)}_{t=t_{0}}^{t_{K}}\right)\propto \text{exp}\left[-\frac{N}{2}\int_{t_{0}}^{t_{K}}dt\gamma s^{⊤}s\right]$$

Larger values of γ penalize large selection coefficients more heavily, shrinking estimates toward zero unless they are well-supported by data.

Second, we introduce another biologically realistic constraint: environmental changes (and consequently selection pressures) typically do not fluctuate arbitrarily quickly. We implement this through a Gaussian prior distribution for the time derivative of the selection coefficients with mean zero and variance $1/N\gamma^{\prime }$,

$$P_{\text{prior}}\left((\dot{s}(t){)}_{t=t_{0}}^{t_{K}}\right)\propto \text{exp}\left[-\frac{N}{2}\int_{t_{0}}^{t_{K}}dt\gamma^{\prime }{\dot{s}}^{⊤}\dot{s}\right]$$

This term penalizes abrupt changes in the s(t), with $\gamma^{\prime }$ determining the expected smoothness in time. Larger values of $\gamma^{\prime }$ are consistent with a gradually changing environment, while smaller values allow for more rapid fluctuations.

Combining the likelihood of the evolutionary dynamics and the prior constraints, we can derive an expression for the selection coefficients $\overset{ˆ}{s}(t)$ that best explain an observed evolutionary trajectory. This maximum *a posteriori* (MAP) estimate is given by the solution of the Euler-Lagrange equation (Supplementary Information)

(2) $$\gamma^{\prime }\ddot{s}=[C(x)+\gamma I]s-[\dot{x}-F(x)-R(x)].$$

The vector $x(t)=\left(x_{1}(t),x_{2}(t),\ldots ,x_{\ell }(t)\right)$ represents the mutant frequency at each site in the genome across the population at time t,

$$x_{i}\left(t\right)=\sum_{a=1}^{M}g_{i}^{a}z_{a}\left(t\right).$$

In (2), I is the identity matrix and C(x(t)) is the mutant frequency covariance matrix, with entries

$$C_{ij}(x(t))=\left\{\begin{array}{ll} x_{i}(t)\left(1-x_{i}(t)\right) & i=j\\ x_{ij}(t)-x_{i}(t)x_{j}(t) & i\neq j \end{array},\right.$$

where $x_{ij}(t)=\sum_{a=1}^{M}g_{i}^{a}g_{j}^{a}z_{a}(t)$. In population genetics, the covariance between mutant frequencies is referred to as linkage disequilibrium. Finally, F(x(t)) and R(x(t)) quantify instantaneous change in mutant frequencies due to spontaneous mutations and recombination, respectively. In the model described above, $F_{i}(x(t))=\mu \left(1-2x_{i}(t)\right)$ and R(x(t))=0. The contribution from recombination is typically nonzero in more complex fitness models that include cooperative effects of mutations at multiple sites in the genome^29,30^.

### Interpreting the Euler-Lagrange equation

Equation (2) provides an explicit expression linking evolutionary dynamics to evidence for a shifting fitness seascape. Under the diffusion approximation of the WF model, the expected instantaneous change in the mutant frequencies, $\dot{x}(t)$, is given by (Supplementary Information)

(3) $$\left\langle \dot{x}\right\rangle =C\left(x\right)\cdot s+F\left(x\right)+R\left(x\right).$$

Thus, if the s(t) are totally unconstrained ($\gamma =\gamma^{\prime }=0$), the most likely selection coefficients would simply be those that exactly reproduce the observed changes mutant frequencies at each moment in time. However, this approach would yield noisy and biologically implausible results.

**Fig. 1.**
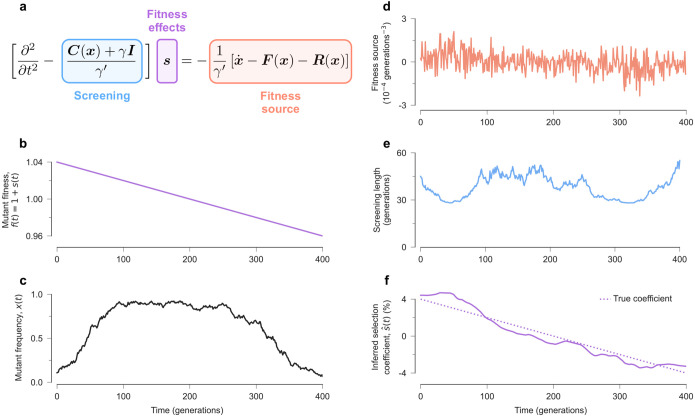
From temporal data to fitness seascapes. **a**, The time-varying fitness effects of mutations that best fit a particular data set, $\overset{ˆ}{s}(t)$, obey a screened Poisson equation. **b**, As an example, we consider a simple model with a single site (ℓ=1), where the fitness effect of mutations shifts from beneficial to deleterious over time. **c**, In an example simulation, mutant frequencies rise and then fall following changes in fitness. Instantaneous changes in mutant frequencies are a fitness “source” that provide evidence of natural selection (**d**), which propagate over a range of time determined by the screening length (**e**). f, Collectively, these terms guide our inference of natural selection over time, which closely follows the true, underlying seascape.

In reality, environmental changes and the resulting shifts in selection often occur gradually (captured by $\gamma^{\prime }>0$). This implies that changes in mutant frequencies reveal information about selection not only at that time point, but also at times in the near past and future. To see this relationship more clearly, we can rearrange (2) as follows (Fig. 1a):

(4) $$\left[\frac{\partial^{2}}{\partial t^{2}}-\frac{C(x)+\gamma I}{\gamma^{\prime }}\right]s=-\frac{1}{\gamma^{\prime }}[\dot{x}-F(x)-R(x)].$$

Interpreting time as a spatial coordinate, the Euler-Lagrange equation has the same form as the screened Poisson equation,

(5) $$\left[\Delta -\frac{1}{\lambda^{2}}\right]\phi \left(r\right)=-\rho \left(r\right),$$

which arises in the context of electrostatic screening and granular flow^31,32^, among other examples.

In the electrostatic context, (5) describes how electric fields are screened in a material with mobile charges. Here, ϕ(r) represents the electric potential, ρ(r) is the source term, and r are spatial coordinates. λ describes the characteristic distance over which screening occurs.

We can therefore provide an intuitive understanding of (4) by relating this expression with the screened Poisson equation in electrostatics (see Fig. 1 for an example). In this analogy, the shape of the fitness seascape is defined by the selection coefficients in time, s(t), which play the role of an electric potential in space, ϕ(r). The fitness seascape drives population evolution, as shown in (3). Changes in mutant frequency, $\dot{x}$, which are not explained by spontaneous mutations or recombination, F(x)+R(x), are like charges that provide evidence of natural selection. Intuitively, beneficial mutations are likely to increase in frequency while deleterious ones decline. The screening length λ is proportional to $\sqrt{\gamma^{\prime }}$, such that the effective time over which frequency changes are informative about fitness depends on how rapidly the environment changes.

The mutant frequency covariance matrix C(x) plays multiple roles in (4). In the simplest case, one can imagine a diagonal covariance matrix, such that the mutations at each site in the genome are uncorrelated. In population genetics, this is referred to as linkage equilibrium. The mutant frequency variance $C_{ii}\left(x_{i}(t)\right)$ is then inversely proportional to the screening length for $s_{i}$. Thus, frequency changes near the boundaries ($x_{i}(t)$ near zero or one) propagate evidence of natural selection further in time.

The off-diagonal terms of C(x) describe how correlations between mutations affect the response to natural selection. Expanding the first term on the right hand side of (3) into diagonal and off-diagonal contributions, one can see that changes in mutant frequencies are driven not just by the fitness effect of the mutation, but also by the net fitness effects of the genetic background. For example, even if a mutation at some site i has no effect on fitness ($s_{i}(t)=0$), we would expect ${\dot{x}}_{i}(t)$ to be positive if mutations at site i were correlated with a beneficial mutation at another site $j\left(C_{ij}(x(t))=x_{ij}(t)-x_{i}(t)x_{j}(t)>0\right.\;\text{and}\;\left.s_{j}>0\right)$.

### Treatment of boundary conditions

We applied the domain extension method to address boundary conditions in the screened Poisson equation. This approach involves expanding the original domain Ω to a larger extended domain $\tilde{\Omega }$, imposing new boundary conditions on $\tilde{\Omega }$, setting the source term to zero in $\tilde{\Omega }$, and using continuity conditions to connect solutions across domains. This approach is especially helpful for preventing artefacts at the edges of the observed time window. We extended the time domain and imposed a transversality condition $\dot{s}\left({\tilde{t}}_{i}\right)=\dot{s}\left({\tilde{t}}_{e}\right)=0$, where ${\tilde{t}}_{i}$ and ${\tilde{t}}_{e}$ denote the initial and end times in the extended domain $\tilde{\Omega }$, respectively (Supplementary Information). The transversality condition in our model is analogous to a Neumann boundary condition in electrostatics, which typically implies no flux through the boundary of an isolated or insulated domain. In our evolutionary context, this condition specifies that the environment is stationary at both the beginning and end of the extended time domain, rather than changing abruptly at the boundaries.

### Validation in simulations

**Fig. 2.**
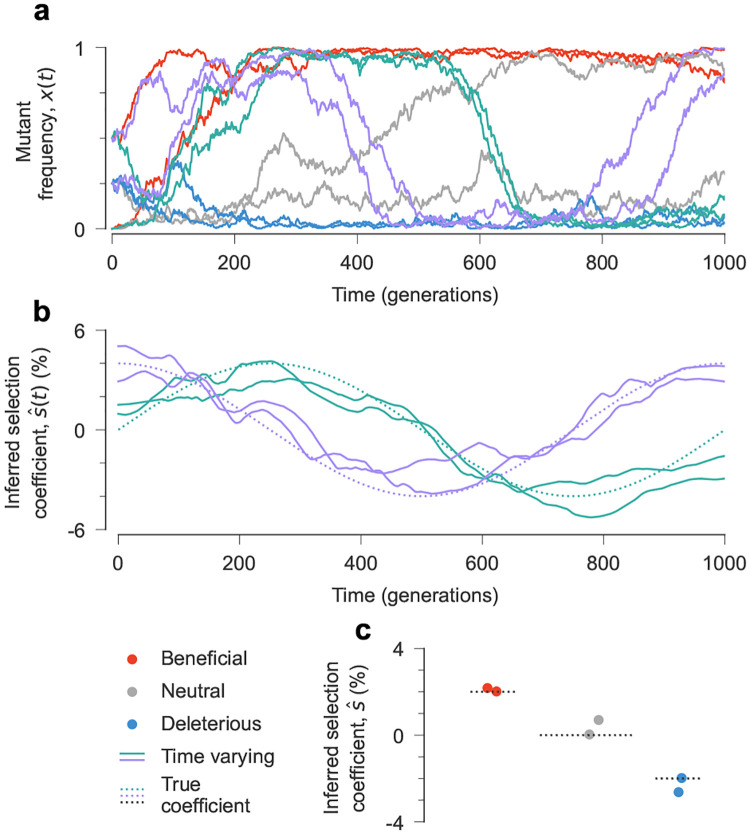
Inference of a simple fitness seascape. **a,** In simulated population dynamics, mutant frequencies change over time, driven by the fitness seascape. The inferred selection coefficients are close to the true, underlying ones for both time-varying parameters (**b**) and constant ones (**c**). Simulation parameters: ℓ=10 sites with two states at each site (mutant and wild type, WT), 2 beneficial mutations with s=0.02, 2 neutral mutations with s=0, and 2 deleterious mutations with s=-0.02. Four selection coefficients follow a sine or cosine pattern over time. Population size $N=10^{3}$, mutation rate $\mu =1\times 10^{-3}$ per site per generation, recombination probability $r=1\times 10^{-3}$ per site per generation. The initial population was randomly generated and evolved over T=1000 generations.

To validate our method, we simulated population evolution on a simple, additive fitness seascape (Fig. 2a). Our model included $N=10^{3}$ individuals with genomes of length ℓ=10. Mutations at six sites had fitness effects that were constant in time: two beneficial, two neutral, and two deleterious, with s(t)=2%,0,-2%, respectively. Mutations at four sites had time-varying effects on fitness: two each with selection coefficients of s(t)=Asin(2πt/τ) and s(t)=Acos(2πt/τ), with amplitude A=4% and period τ=1000 generations. Our simulations included mutations and recombination with rates $\mu =r=1\times 10^{-3}$ per site per generation.

**Fig. 3.**
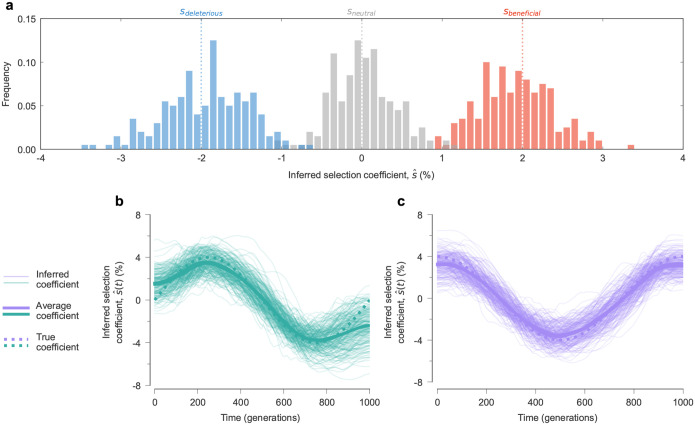
Robustness of inference across multiple replicate simulations. **a,** Distribution of constant selection coefficients inferred by our approach from 100 replicate simulations using the same parameters as in Fig. 2. **b, c**, The inferred trajectories of time-varying selection coefficients in individual simulations, and their average values, are close to the true ones.

Figure 2b–c displays results from a typical simulation, showing that our method can distinguish between beneficial, neutral, and deleterious mutations with constant effects and accurately estimate fluctuating selection. We performed 100 independent simulations to verify the consistency of our results (Fig. 3). We further tested the robustness of our approach to model misspecification, i.e., incorrectly assuming time-varying selection at sites where selection is actually constant. Supplementary Fig. S1 shows that, even with this incorrect assumption, our method recovers selection coefficients that fluctuate around the true, constant values rather than producing spurious patterns.

We also systematically explored how different values of the temporal smoothness parameter $\gamma^{\prime }$ affect our inference. We focused in particular near the boundaries of the observation window, where our inferences are less constrained by data (Supplementary Fig. S2; Supplementary Information). These tests helped us to identify appropriate choices for $\gamma^{\prime }$ that balance between overly rigid constraints (which might mask true fluctuations) and insufficient regularization (which could lead to spurious fluctuations, especially near the data boundaries).

### Fluctuating fitness shapes HIV-1 evolution

**Fig. 4.**
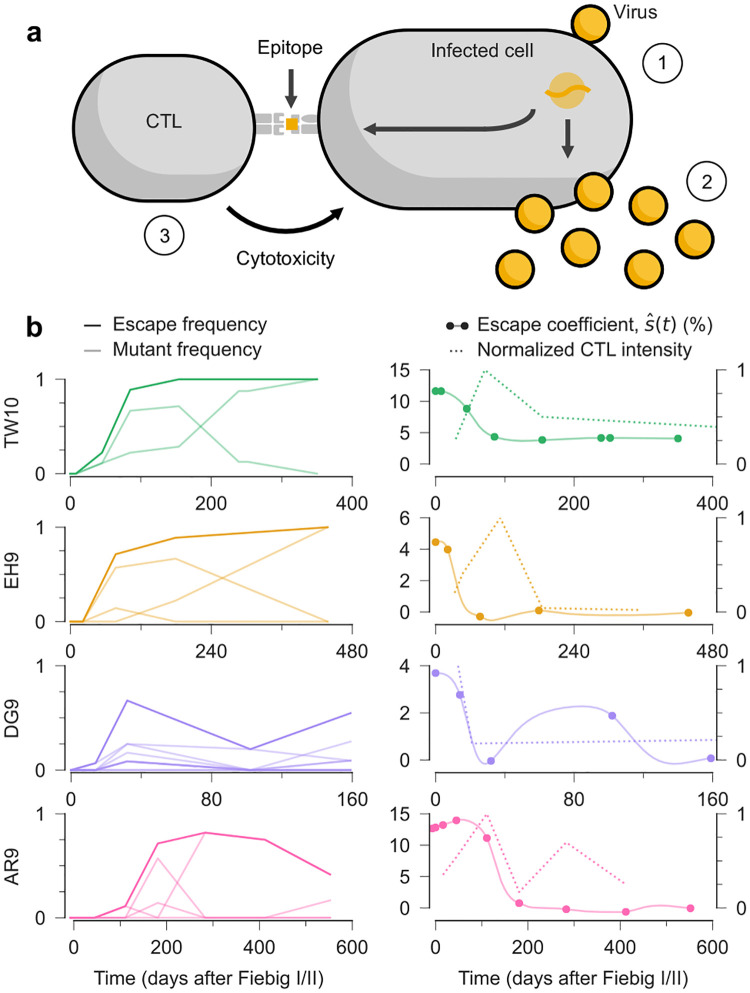
Estimates of time-varying selection for CTL escape. **a**, Schematic representation of HIV-1 infection and immune control. Viruses enter vulnerable cells (1) and replicate, producing new viral particles that can spread infection (2). However, CTLs can recognize viral epitopes presented on the infected cell surface, triggering the release of cytotoxic granules that kill the infected cell and cut short viral replication (3). **b**, Frequency of viruses with one or more escape mutations (escape frequency) and the frequency of individual escape mutations over time for four example CTL epitopes. Time is measured in days after Fiebig stages I/II, an early acute phase of HIV-1 infection roughly 1–2 weeks after transmission^52^. The fitness benefit of escape that we infer (escape coefficient, $\overset{ˆ}{s}(t)$) varies over time, peaking before the experimentally measured peak of the CTL response. For each epitope, CTL responses were normalized by dividing the measured spot forming units per million peripheral blood mononuclear cells (SFU/10^6^ PBMCs) by its maximum value.

We applied our inference framework to analyze evolutionary data from HIV-1, focusing on viral adaptation within infected individuals. HIV-1 provides an ideal test case for our method because it evolves rapidly under strong, dynamical selection pressures from the host immune system. Although the immune response to HIV-1 is multifaceted, cytotoxic T lymphocytes (CTLs, also known as $\text{CD}8^{+}$ “killer” T cells) play an especially important role in controlling viral replication^33^. CTLs recognize and eliminate HIV-1-infected cells through a highly specific molecular mechanism (see Fig. 4a). Viral proteins inside infected cells are broken down into short peptide fragments, called epitopes, that are displayed on the cell surface by MHC molecules. CTLs can use their T cell receptors to detect these viral epitopes. This triggers the release of cytotoxic molecules that kill the infected cell, thereby limiting viral replication.

However, HIV-1 can escape immune control through mutations in CTL epitopes. Due to the virus’s high rate of mutation and replication^34,35^, HIV-1 frequently acquires “escape mutations” that prevent CTL recognition while allowing the virus to continue replicating^33,36–38^. This creates an evolutionary arms race, with HIV-1 evolution driven by the pressure to escape from dynamic immune responses.

Prior work has shown that the evolutionary pressure to escape CTLs is strong^26,39–41^ but difficult to quantify precisely^41–43^. A particularly puzzling observation is that viral escape from CTL responses that emerge later in infection tends to occur more slowly than escape from early responses^44–46^. Several mechanisms have been proposed to explain this pattern, including competition between multiple escape variants (clonal interference)^47^, greater fitness costs for escape mutations in later epitopes^45^, and an increase in the breadth immune response^48^. Here, we study how the fitness benefit of T cell escape shifts over time, compared with the dynamics of the immune response.

To capture the key biological features of immune escape, we extended our fitness model to differentiate between two types of selection pressures. First, we defined time-varying escape coefficients that quantify the fitness effect of having one or more mutations within a CTL epitope^49^ (Supplementary Information). These coefficients capture the advantage of immune evasion. Second, we defined selection coefficients that quantify the effects of specific mutations (both within and outside CTL epitopes) on intrinsic viral replication, independent from immune evasion. We allowed escape coefficients to vary over time, reflecting changing immune pressures, while selection due to individual mutations was held constant. This follows from the biological intuition that immune selection likely fluctuates much more rapidly than other selective forces during infection. Tests in simulations confirmed our ability to distinguish these different fitness effects and track their changes over time (Supplementary Figs. S3–S5).

After validating our method in simulations, we applied it to longitudinal HIV-1 sequence data from 13 individuals^50^. This data set offered several advantages. Donors were followed from early infection up to a few years before initiating antiretroviral drug therapy, capturing a critical period of immune-virus coevolution. Regular blood samples provided snapshots of the HIV-1 population over time as well as measurements of each individual’s immune response. In addition, the absence of antiretroviral drugs meant that immune pressure was the dominant force of selection guiding viral evolution. For our analysis, we incorporated HIV-1-specific rates of mutation^51^ and recombination^22^ to accurately model the evolution of the virus (Supplementary Information). To reduce confounding factors, we focused on 37 CTL epitopes where the fitness effect of immune escape could be statistically separated from the fitness effects of the underlying escape mutations and genetic background^49^ (Supplementary Information). For 32 of these, the frequency and intensity of CTL responses against each epitope were also measured in enzyme-linked immunospot (ELISpot) assays^50^. This allowed us to directly compare our estimates of selection with independent, experimentally measured immune activity.

#### CTL intensity and the fitness effect of immune escape

Our analysis revealed a striking temporal pattern: for more than 90% of the CTL epitopes studied, the inferred fitness advantage of immune escape peaked at or before the maximum intensity of the corresponding CTL response (Fig. 4b, Supplementary Figs. S6–S7). This counterintuitive finding, where the selective advantage of CTL escape declines even as the immune response appears to become stronger, suggests an unappreciated complexity in HIV-1-immune dynamics.

This pattern could result from T cell exhaustion, a phenomenon in chronic viral infections where persistently stimulated T cells gradually lose their functional capabilities^53–55^. T cell functions decline in a stepwise manner during exhaustion, and cytotoxicity is one of the first functions to be impaired^53,54^. In contrast, secretion of the cytokine interferon gamma, measured by the ELISpot assays used in this study^50^, is one of the CTL functions that is most resistant to exhaustion^53,54^. Our findings therefore suggest that the actual cytotoxic pressure on the virus may have already begun declining by the time that cytokine production peaks. This could explain why the fitness benefit of escape decreases even while the strength of the immune response appears to increase.

#### Kinetics of CTL escape

The evolutionary trajectories we observed varied significantly across different epitopes. For the majority of epitopes, we observed straightforward escape dynamics: once escape mutations appeared, they rapidly increased in frequency and remained dominant in the viral population for the duration of observations. However, a subset of epitopes exhibited more complex, non-monotonic patterns where escape variant frequencies fluctuated over time. Notable examples include the DG9 and AR9 epitopes (Fig. 4b), where escape frequencies increased initially before declining later in infection. The fitness benefit of escape that we infer reflects these dynamics, rising and falling along with escape mutants.

#### Long-term decline in the fitness benefit of immune escape

Beyond the relationship with measured CTL responses, our analysis revealed a consistent long-term trend: the fitness advantage of immune escape declined substantially over the course of infection across nearly all epitopes (Supplementary Fig. S8). For many epitopes, selection for immune escape appears to be minimal a year or more after infection. This observation is aligned with previous observations of decreasing escape rates over time^44–46^ but provides a more direct quantification of the underlying selection pressures. Because our model accounts for the intrinsic fitness effects of escape mutations, competition between escape variants, and correlations with mutations in the genetic background, these factors are unlikely to explain the declining benefit of immune escape.

However, our findings are consistent with the hypothesis of progressive immune dysfunction. In chronic infection, HIV-1-specific CTLs can become increasingly exhausted and lose their ability to effectively control viral replication. A reduction in CTL killing ability would also reduce selection for escape. This may explain why HIV-1 escape variants sometimes stall or even decline in frequency despite continued detection of the corresponding CTL response.

## Discussion

Evolution is guided by the shifting constraints of natural selection. Accurately quantifying these selection pressures represents both a major challenge and opportunity for evolutionary biology, with applications ranging from understanding adaptation in natural populations to guiding experimental evolution. Here, we developed a novel theoretical approach to infer a time-varying fitness seascape from genetic sequence data. Our approach is based on a path integral method from statistical physics, where “paths” represent histories of population evolution. Applying Bayesian inference methods, we found that the fitness effects of mutations that best explain an observed evolutionary history obey an Euler-Lagrange equation with the same mathematical form as the screened Poisson equation.

This connection to physics provides an intuitive interpretation. In our framework, changes in mutant frequencies that cannot be explained by mutation or recombination act as “charges” that reveal the underlying fitness seascape, analogous to the association between electric charges and the electric potential. The screening length in our equation determines how far in time these “charges” influence inferred selection, allowing us to distinguish between rapid fluctuations and more gradual shifts in fitness. Through extensive simulations, we confirmed that our method accurately recovers both constant and time-varying selection.

Applying our framework to longitudinal HIV-1 sequence data uncovered unexpected host-pathogen dynamics. We discovered that the fitness advantage of immune escape peaks at or before the maximum intensity of CTL responses, as measured by cytokine secretion assays. We also observed a consistent pattern of declining selection for immune escape over time across nearly all epitopes. This occurred despite the continued detection of HIV-1-specific CTL responses. Collectively, these observations are consistent with a model of progressive immune exhaustion in chronic HIV-1 infection. Chronically stimulated CTLs appear to lose their ability to control viral replication, weakening selection for immune escape. Our results highlight the dynamic interplay between viral evolution and immunity, showing that the fitness constraints guiding HIV-1 evolution can shift over time even within a single individual.

While our findings align with and extend prior work showing a reduced rate of immune escape late in infection^44–46^, several limitations of the data should be considered when interpreting our results. Viral sequence data has inherent constraints. The limited number of sequences per time point (typically 5–20) and sometimes irregular sampling intervals introduce uncertainty in the true evolutionary trajectories. In turn, this increases the uncertainty in the fitness effects that we infer. CTL intensities were also sparsely sampled in time, making it difficult to track their dynamics precisely. Despite these challenges, the consistency of the relationship that we observe between selection for immune escape and CTL intensity supports the robustness of our results.

Future work could improve our inference framework. One limitation of our approach is that we treat the observed mutant frequencies (and their correlations) as exact, when in reality they are affected by finite sampling. Recent studies have developed clever methods to sample possible mutant frequency trajectories that are consistent with finitely-sampled data^8,56^. Explicitly modeling sampling uncertainty could also improve the robustness of our results. This approach would be especially helpful for dealing with data that are sparsely sampled in time. Currently, we use linear interpolation between observed time points, which can occasionally introduce artefacts in inferred selection coefficients (see Supplementary Fig. S9). Though these effects appear relatively minor in our HIV-1 analysis, a more sophisticated approach that incorporates both sampling uncertainty and biologically plausible frequency trajectories between observations could yield even more robust inferences.

Overall, our work introduces a flexible theoretical framework for inferring fluctuating fitness seascapes from temporal genetic data. While demonstrated here using HIV-1 evolution, our method could be applied to study evolution in other biological contexts. Our framework can also readily be extended to incorporate more complex fitness functions (as shown in our analysis of HIV-1 immune escape) or features such as environmental covariates and spatial structure. This generality could enable new insights into diverse evolutionary phenomena in both experimental and natural populations. The ability to quantify how fitness landscapes shift over time represents an important step toward a more complete understanding of evolutionary processes in changing environments.

## Supplementary Material

1
