# Supplementary material for: Inferring fitness seascapes from evolutionary histories
Source: bioRxiv. 2025 Jun 8:2025.06.08.658500. Preprint. [Version 1] doi: 10.1101/2025.06.08.658500 (PMC12259008; doi:10.1101/2025.06.08.658500)
Supplement: 1 [file NIHPP2025.06.08.658500V1-supplement-1.pdf]

bioRxiv preprint doi: https://doi.org/10.1101/2025.06.08.658500; this version posted June 9, 2025. The copyright holder for this preprint (which was not certified by peer review) is the author/funder, who has granted bioRxiv a license to display the preprint in perpetuity. It is made available under aCC-BY 4.0 International license.

Supplementary Information

Evolutionary model

In our study, we model the evolution of a population of N individuals subject to recombination, mutation, and natural selection, following the Wright-Fisher (WF) model. For simplicity, we first assume that each site can be in either a wild-type (WT) or mutant state. In population genetics, the terms "locus" and "allele" are often used to describe locations in the genome and the state of the genetic sequence there, respectively. For consistency with the evolutionary biology literature, we will use the same terminology. Individuals possess a genetic sequence length of ℓ, resulting in a total of $M = 2^\ell$ possible genotypes. We further assume that there exist λ binary traits, which depend on the presence or absence of mutant alleles at specific sites and are subject to selection.

Let $n_a(t_k)$ represent the number of individuals with genotype a at generation t_k, and define $z_a(t_k) = n_a(t_k)/N$ as the frequency of genotype a at generation t_k. Thus, the state of the population at generation t_k can be defined by the vector $z(t_k) = (z_a(t_k), z_b(t_k), \ldots, z_M(t_k))$.

In our fitness model, a positive selection coefficient h_a for genotype a indicates that this genotype has higher fitness compared to the wild type, whose fitness is set to 1. As in the constant inference case[57], we divide the fitness of each genotype into two parts: individual alleles (quantified by selection coefficients s_i) and binary traits (quantified by trait coefficients s_n). In the context of HIV-1 evolution, binary traits represent CTL escape: a virus either has mutations within an epitope, which we assume allows the virus to escape immunity, or it does not. We justify this approximation because the binding of T cell receptors to viral epitopes is highly specific[57]. These traits could represent different biological features in other contexts. The fitness effects of different mutant alleles and binary traits are additive,

$$f_a = 1 + h_a = 1 + \sum_i^{\ell} s_i g_i^a + \sum_n^{\lambda} s_n g_n^a \,. \tag{S1}$$

Here g is an indicator function to determine the presence of a mutation in locus i or trait n. We represent the haploid genetic sequence of each individual a as a binary string $g^a = (g_1^a, g_2^a, \ldots, g_\ell^a, g_{n_1}^a, g_{n_2}^a, \ldots, g_{n_\lambda}^a)$ with $g_i^a \in \{0,1\}$. Here the index i labels each site in the genetic sequence, running from 1 to ℓ; the index n_i labels each binary trait, running from n_1 to n_λ; and a labels the genotype (of which there are $M = 2^\ell$ possibilities). The effects of mutant alleles at different loci are cumulative, meaning that more beneficial alleles result in higher fitness. The fitness effects of different traits are also additive. In principle both the trait coefficients s_n and selection coefficients s_i can be functions of time, though we fix the selection coefficients s_i for our analysis of HIV-1 data.

The WF model also incorporates recombination and mutation. For simplicity, we assume that the recombination rate r and mutation rate μ are constant (though later we will relax this assumption). The mutation rate μ is the probability that the wild-type at one site converts to the mutant state, or vice versa. Given the exceedingly low mutation rate μ, we assume that each sequence undergoes at most one mutation per generation. Recombination refers to the process of genetic exchange between individuals, producing a "child" genome with shuffled contributions from each of the "parents." We write the probability of a recombination breakpoint per site per reproduction cycle as r.

After accounting for the effects of selection, mutation, and recombination, the expected frequency of genotype a at generation t_{k+1}, denoted as $p_a(t_{k+1})$, is

$$p_a(z(t_k)) = \frac{y_a(t_k) f_a + \mu \sum_{b|d(a,b)=1} \left[y_b(t_k) f_b - y_a(t_k) f_a \right]}{\sum_{b=1}^{M} y_b(t_k) f_b} \,,$$

$$y_a(t_k) = (1-r)^{\ell-1} z_a(t_k) + (1-(1-r)^{\ell-1}) \psi_a(t_k) \,, \tag{S2}$$

$$\psi_a(t_k) = \sum_c^M \sum_d^M R_{a,cd} z_c(t_k) z_d(t_k) \,. \tag{S3}$$

The notation $b|d(a,b) = 1$ indicates that genotypes a and b differ by just a single mutation. Under our assumption that each sequence undergoes at most one mutation each generation, genotypes b encompasses all genotypes that genotypes a can mutate to within one generation. The term $\psi_a(t_k)$ represents the probability that the random recombination of any two individuals (c and d) within the population produces an offspring of genotype a, including cases where both parent and offspring share the same genotype a. Under WF dynamics, the probability of observing genotype frequencies $\boldsymbol{z}(t_{k+1})$ at the next generation t_{k+1}, given genotype frequencies of $\boldsymbol{z}(t_k)$= $(z_a(t_k), z_b(t_k), \ldots, z_M(t_k))$ at generation t_k, is multinomial:

$$P(\boldsymbol{z}(t_{k+1}) | \boldsymbol{z}(t_k)) = N! \prod_{a=1}^{M} \frac{(p_a(t_{k+1}))^{N z_a(t_{k+1})}}{(N z_a(t_{k+1}))!} \,. \tag{S4}$$

bioRxiv preprint doi: https://doi.org/10.1101/2025.06.08.658500; this version posted June 9, 2025. The copyright holder for this preprint (which was not certified by peer review) is the author/funder, who has granted bioRxiv a license to display the preprint in perpetuity. It is made available under aCC-BY 4.0 International license.

Consequently, given the selection and trait coefficients $\boldsymbol{s}$, the likelihood that the genotype frequency vector follows a specific evolutionary trajectory, $(\boldsymbol{z}(t_1), \boldsymbol{z}(t_2), \ldots, \boldsymbol{z}(t_K))$, is

$$P(\boldsymbol{z}(t_k)_{t_0}^{t_K}|\boldsymbol{s}) = \prod_{k=0}^{K-1} P(\boldsymbol{z}(t_{k+1})|\boldsymbol{z}(t_k)) \,. \tag{S5}$$

This complex likelihood function is conditioned upon the initial state $\boldsymbol{z}(t_0)$. Fortunately, (Eq. S4) can be simplified by using a diffusion approximation[58].

Diffusion approximation

Since the populations are large and the changes in allele frequencies between generations are relatively small, we can transform the discrete, generation-by-generation model to a continuous-time model in the limit

$$N \to \infty, \text{ with } \boldsymbol{s}, \mu, r \text{ of } \mathcal{O}(1/N) \,.$$

Given that the above parameters are of first-order infinitesimals ($\mathcal{O}(1/N)$), and dropping second-order and higher infinitesimals, $p_a(t)$ (S2) can be written as

$$\begin{aligned} p_a(z(t)) &= z_a(t) + z_a(t)\left(h_a - \sum_{b=1}^{M} h_b z_b(t)\right) + \mu\left(-L z_a(t) + \sum_{b|d_{ab}=1}^{M} z_b(t)\right) \\ &\quad - r(L-1)(z_a(t) - \psi_a(z(t))) + \mathcal{O}\left(\frac{1}{N^2}\right) \\ &= z_a(t) + \mathcal{O}\left(\frac{1}{N}\right), \end{aligned}$$

Preliminary expansions for $\Delta t = 1$

Since the Wright-Fisher model is a Markov process, we can use the following the conditional moment generating function

$$M(\boldsymbol{f}, \boldsymbol{z}(t)) = \left(\sum_{a=1}^{M} p_a(\boldsymbol{z}(t)) \exp\left(\frac{f_a}{N}\right)\right)^N$$

to obtain the following physical quantities:

$$\begin{aligned} \mathbb{E}[z_a(t+1)] &= \left.\frac{\partial M}{\partial f_a}\right|_{\boldsymbol{f}=0} = p_a(\boldsymbol{z}(t)) \,, \\ \mathbb{E}\left[z_a^2(t+1)\right] &= \frac{p_a(t)}{N} + \frac{(N-1)}{N} p_a^2(t) \\ \mathbb{E}\left[z_a^3(t+1)\right] &= \frac{(N-1)(N-2)}{N^2} p_a^3(t) + \frac{3(N-1)}{N^2} p_a^2 + \frac{1}{N^2} p_a(t) \\ \mathbb{E}[z_a(t+1) z_b(t+1)] &= \frac{(N-1)}{N} p_a(t) p_b(t) \\ \mathbb{E}[z_a(t+1) z_b(t+1) z_c(t+1)] &= \frac{(N-1)(N-2)}{N^2} p_a(t) p_b(t) p_c(t) \,. \end{aligned}$$

Based on these results, after neglecting second-order and higher infinitesimals, the first-order (drift), second-order (diffusion), and third-order terms for genotype frequency changes at $\Delta t = 1$ are given by:

$$\begin{aligned} \mu_1 &= \mathbb{E}[z_a(t+1) - z_a(t)] = p_a(t) - z_a(t) + \mathcal{O}\left(\frac{1}{N^2}\right) \\ \mu_2 &= \mathrm{Var}[z_a(t+1)] = \mathbb{E}\left[z_a^2(t+1)\right] - (\mathbb{E}[z_a(t+1)])^2 = \frac{z_a(t)(1 - z_a(t))}{N} + \mathcal{O}\left(\frac{1}{N^2}\right) \\ M_{ab}^{(2)} &= \mathrm{Covar}[z_a(t+1), z_b(t+1)] = \mathbb{E}[z_a(t+1) z_b(t+1)] - \mathbb{E}[z_a(t+1)]\,\mathbb{E}[z_b(t+1)] = -\frac{z_a(t) z_b(t)}{N} + \mathcal{O}\left(\frac{1}{N^2}\right), \\ \mu_3 &= \mathbb{E}\left[(z_a(t+1) - \mathbb{E}[z_a(t+1)])^3\right] = \mathcal{O}\left(\frac{1}{N^2}\right), \\ M_{abc}^{(3)} &= \mathbb{E}[(z_a(t+1) - \mathbb{E}[z_a(t+1)])(z_b(t+1) - \mathbb{E}[z_b(t+1)])(z_c(t+1) - \mathbb{E}[z_c(t+1)])] = \mathcal{O}\left(\frac{1}{N^2}\right). \end{aligned}$$

bioRxiv preprint doi: https://doi.org/10.1101/2025.06.08.658500; this version posted June 9, 2025. The copyright holder for this preprint (which was not certified by peer review) is the author/funder, who has granted bioRxiv a license to display the preprint in perpetuity. It is made available under aCC-BY 4.0 International license.

Main derivations for arbitrary Δt

To obtain the value at arbitrary Δt, we incrementally advance t from 1 to Δt in unit steps and apply mathematical induction to establish the result.

$$\begin{aligned}\mathbb{E}[z_a(\mathbf{z}(t+2))] &= p_a(\mathbf{z}(t+1)) = p_a(\mathbf{p}(\mathbf{z}(t)))\\ &= p_a(\mathbf{z}(t)) + p_a(\mathbf{z}(t))h_a + \mu\left(-Lp_a(\mathbf{z}(t)) + \sum_{b|d_{ab}=1}^{M} p_b(\mathbf{z}(t))\right)\\ &\quad -\sum_{b=1}^{M} h_b\mathbb{E}[p_a(t)p_b(t))] - r(L-1)\left(p_a(\mathbf{z}(t)) - \mathbb{E}[\psi_a(t+1)]\right)\end{aligned}$$

Here ψ_a is defined in S3. By applying the law of total expectation repeatedly, we can get the remaining expectations.

$$\begin{aligned}\mathbb{E}[p_a(t)p_b(t))] &= z_a(t)z_b(t)) + \mathcal{O}\left(\frac{1}{N}\right)\\ \mathbb{E}[\psi_a(\mathbf{z}(t+1))] &= \sum_{c=1}^{M}\sum_{d=1}^{M} R_{a,cd}\mathbb{E}[z_c(t+1)z_d(t+1)] = \sum_{c=1}^{M}\sum_{d=1}^{M} R_{a,cd}(N-1)p_cp_d/N\\ &= \sum_{d=1}^{M} R_{a,cd}z_c(t)z_d(t) + \mathcal{O}\left(\frac{1}{N}\right) = \psi_a(\mathbf{z}(t)) + \mathcal{O}\left(\frac{1}{N}\right)\end{aligned}$$

Thus,

$$\begin{aligned}\mathbb{E}[z_a(\mathbf{z}(t+1))] &= z_a(t) + 1(p_a(\mathbf{z}(t)) - z_a(t))\\ \mathbb{E}[z_a(\mathbf{z}(t+2))] &= z_a(t) + 2(p_a(\mathbf{z}(t)) - z_a(t)) + \mathcal{O}\left(\frac{1}{N^2}\right),\\ \mathbb{E}[z_a(\mathbf{z}(t+\Delta t))] &= z_a(t) + \Delta t(p_a(\mathbf{z}(t)) - z_a(t)) + \mathcal{O}\left(\frac{1}{N^2}\right).\end{aligned}$$

Because of the Markov property of the WF process, we can use the law of total expectation and the law of total variance to get

$$\begin{aligned}\mathbb{E}\left[z_a^2(\mathbf{z}(t+\Delta t))\right] &= z_a^2(t) + \mathcal{O}\left(\frac{1}{N}\right),\\ \mathrm{Var}\left(z_a(t+\Delta t)\right) &= \Delta t\frac{z_a(t)(1-z_a(t))}{N} + \mathcal{O}\left(\frac{1}{N^2}\right),\\ \mathrm{Covar}\left(z_a(t+\Delta t), z_b(t+\Delta t)\right) &= -\Delta t\frac{z_a(t)z_b(t)}{N} + \mathcal{O}\left(\frac{1}{N^2}\right),\\ \mu_3(z_a(t+\Delta t)) &= \mathcal{O}\left(\frac{1}{N^2}\right),\\ M_{abc}^{(3)}(z_a(t+\Delta t), z_b(t+\Delta t), z_c(t+\Delta t)) &= \mathcal{O}\left(\frac{1}{N^2}\right),\end{aligned}$$

Rescaling of time

In our framework, we can consider the scaling limit in which the population size becomes very large ($N \to \infty$), where frequencies transition from discrete values in the set $0, \frac{1}{N}, \frac{2}{N}, \cdots, 1$ to a continuous values in the interval $[0,1]$. We rescale time as $1/N$, with the continuous difference $\delta t = \Delta t/N$. After rescaling of time and frequency, we denote the genotype frequency as $\tilde{z}_a(t)$. Considering Wright-Fisher model is a Markov process, and the increment $\delta\tilde{z}$ of the process during a time interval $[t, t+\delta t]$ satisfies the following conditions:

bioRxiv preprint doi: https://doi.org/10.1101/2025.06.08.658500; this version posted June 9, 2025. The copyright holder for this preprint (which was not certified by peer review) is the author/funder, who has granted bioRxiv a license to display the preprint in perpetuity. It is made available under aCC-BY 4.0 International license.

1. The expected value of $\Delta \check{z}_a = \check{z}_a(t+\delta t) - \check{z}_a(t)$ is proportional to δt.

$$
\begin{aligned}
\mathbb{E}\left[\Delta z_a\right] &= \mathbb{E}\left[\check{z}_a(t+\delta t)\right] - \mathbb{E}\left[\check{z}_a(t)\right] \\
&= N\delta t \left(\check{z}_a(t)\left(h_a - \sum_{b=1}^{M} h_b \check{z}_b(t)\right) + \mu \left(-L\check{z}_a(t) + \sum_{b|d_{ab}=1}^{M} \check{z}_b(t) \right) - r(L-1)(\check{z}_a(t) - \psi_a(\check{z}(t))) \right) + \mathcal{O}\left(\frac{1}{N^2}\right) \\
&= \check{d}_a(t)\delta t + \mathcal{O}\left(\frac{1}{N^2}\right)
\end{aligned}
$$

Here $\check{d}_a(t)$ is known as the drift vector, describing the rate of expected changes in genotype frequencies at time t (see equation 4.100 of Risken[59]). We will discuss its expansion in the next section.

$$
\check{d}_a(t) = \lim_{\delta t \to 0} \frac{\mathbb{E}\left[\Delta z_a\right]}{\delta t} = N(\check{p}_a(t) - \check{z}_a(t))
$$

2. The variance of $\delta \check{z}_a$ and covariance between $\delta \check{z}_a$ and $\delta \check{z}_b$ are also proportional to δt. Here we have

$$
\begin{aligned}
\mathrm{Var}\left(\check{z}_a(t+\delta t)\right) &= \delta t \check{z}_a(t)(1-\check{z}_a(t)) + \mathcal{O}\left(\frac{1}{N^2}\right), \\
\mathrm{Covar}\left(\check{z}_a(t+\delta t), \check{z}_b(t+\delta t)\right) &= -\delta t \check{z}_a(t)\check{z}_b(t) + \mathcal{O}\left(\frac{1}{N^2}\right).
\end{aligned}
$$

These terms are encapsulated in the diffusion matrix $\check{D}_{ab}(t)$, which describes the scaled covariance of the genotype frequency changes (see equation 4.100 of Risken[59]).

$$
\check{D}_{ab}(t) = \begin{cases} \frac{1}{2}\lim_{\delta t\to 0} \frac{\mathrm{Var}(\check{z}_a(t+\delta t))}{\delta t} \approx \check{z}_a(t)(1-\check{z}_a(t))/2 & a = b, \\ \frac{1}{2}\lim_{\delta t\to 0} \frac{\mathrm{Covar}(\check{z}_a(t+\delta t),\check{z}_b(t+\delta t))}{\delta t} \approx -\check{z}_a(t)\check{z}_b(t)/2 & a \neq b. \end{cases}
$$

3. Higher order terms are subleading in $1/N$ and can be omitted.

The random function $\check{z}_a(t)$ is called a diffusion process[58]. The diffusion process is described by the probability density function $\phi(\boldsymbol{z}, t)$,

$$
\frac{\partial \phi}{\partial \tau} = \left[-\sum_{a}^{M} \frac{\partial}{\partial \check{z}_a} \check{d}_a(\tau) + \sum_{a,b}^{M} \frac{\partial^2}{\partial \check{z}_a \partial \check{z}_b} \check{D}_{ab}(\tau) \right] \phi,
$$

which is known as the Fokker-Planck equation.

Path integral likelihood at genotype level

Under this diffusion approximation, using equation 4.109 of Risken[59], the transition probabilities (Eq. S4) become

$$
\begin{aligned}
\phi(\check{\boldsymbol{z}}(t+\delta t)|\check{\boldsymbol{z}}(t)) &= \left[\left(\frac{1}{4\pi\delta t}\right)^{M/2} \cdot \frac{d\check{\boldsymbol{z}}(t+\delta t)}{\sqrt{\det C(\check{z}(t))}} \right] \\
&\times \exp\left[-\sum_{a,b}^{M} \frac{1}{4\delta t} \left[\check{z}_a(t+\delta t) - \check{z}_a(t) - d_a(\check{z}(t))\delta t\right] \left[C_{ab}(\check{z}(t))\right]^{-1} \left[\check{z}_b(t+\delta t) - \check{z}_b(t) - d_b(\check{z}(t))\delta t\right] \right],
\end{aligned}
$$

As noted before, here d_a are terms of the drift vector and $C_{ab}/2N$ are entries of the diffusion matrix. As seen from the definition, the covariance matrix C is a symmetric matrix with length M. At the genotype level, it can be written as:

$$
C_{ab}(z(t)) \approx \begin{cases} z_a(t)(1-z_a(t)) & a = b, \\ -z_a(t)z_b(t) & a \neq b. \end{cases}
$$

bioRxiv preprint doi: https://doi.org/10.1101/2025.06.08.658500; this version posted June 9, 2025. The copyright holder for this preprint (which was not certified by peer review) is the author/funder, who has granted bioRxiv a license to display the preprint in perpetuity. It is made available under aCC-BY 4.0 International license.

The drift vector is more complicated, and we will discuss its expansion later. This term is given by

$$
\begin{aligned}
d_a(z(t)) &= \langle p_a(z(t)) - z_a(t) \rangle \\
&\approx \left(h_a - \sum_b^M h_b z_b(t) \right) z_a(t) + \mu \sum_{b|d(a,b)=1} (z_b(t) - z_a(t)) + r(\ell-1)(z_a(t) - \psi_a(t)) \\
&= \sum_b C_{ab}(t) h_b + \mu \sum_b^M E_{ab} z_b + r(\ell-1)(z_a(t) - \psi_a(t)) \,,
\end{aligned}
\tag{S6}
$$

with the matrix E_{ab} reflecting the differences between genotype a and b,

$$
E_{ab} = \begin{cases} -\ell, & a = b, \\ 0, & d(a,b) > 1, \\ 1, & d(a,b) = 1; \end{cases}
$$

We can then express the likelihood of an evolutionary trajectory (Eq. S5) as a path integral, written in continuous time

$$
P(\boldsymbol{z}|\boldsymbol{z}(t_0)) \propto \exp\left[-\frac{N}{2} \boldsymbol{S}(\boldsymbol{z}(t))_{t_0}^{t} \right]
$$

with the action $\boldsymbol{S}(\boldsymbol{z}(t))_{t_0}^{t_K}$ written as

$$
\boldsymbol{S}(\boldsymbol{z}(t))_{t_0}^{t}) = \int \left\{ [\dot{\boldsymbol{z}}^\intercal(t) - \boldsymbol{d}^\intercal(\boldsymbol{z}(t))][\boldsymbol{C}(\boldsymbol{z}(t))]^{-1}[\dot{\boldsymbol{z}}(t) - \boldsymbol{d}(\boldsymbol{z}(t))] \right\} dt \,.
$$

Here $\dot{\boldsymbol{z}}(t)$ denotes the time derivative of the genotype frequency vector $\boldsymbol{z}(t)$. In this expression, we have also written the frequency change, drift vector, and diffusion matrix in their vector/matrix forms, rather than explicitly writing genotype indices.

Bayesian inference for time-varying selection

To control our estimates, we incorporate prior distributions for the selection and trait coefficients. Here we use a Gaussian distribution with zero mean for the selection coefficients $\boldsymbol{s}$. This approach helps curb the inference of strong fitness effects in the absence of strong statistical evidence:

$$
\begin{aligned}
P_{\text{prior}}(\boldsymbol{s}) &= \frac{1}{(2\pi\sigma^2)^{(\ell+\lambda)/2}} \exp\left(-\int \frac{1}{2\sigma^2} \boldsymbol{s}^\intercal(t) \boldsymbol{s}(t) dt \right) \\
&\propto \exp\left(-\frac{N}{2} \int \gamma \boldsymbol{s}^2(t) dt \right) \,.
\end{aligned}
$$

The maximum *a posteriori* estimate for the selection coefficients can then be found by maximizing the action S, similar to the case where selection coefficients are constant. However, this approach results in a problematic estimator that is extremely noisy with respect to time. This is because the estimates for $\boldsymbol{s}$ at different times are independent from one another, allowing estimates for successive times to vary dramatically. To address this issue, we add a Gaussian prior for the time derivative of the selection coefficients $\dot{\boldsymbol{s}}$, also with zero mean and $1/(N\gamma')$ variance. Combining these two prior distributions, we have:

$$
P_{\text{prior}}(\boldsymbol{s}(t)) \propto \exp\left[-\frac{N}{2} \int \left(\gamma \boldsymbol{s}^2(t) + \gamma' \dot{\boldsymbol{s}}^2(t) \right) dt \right] \,.
$$

This prior implies that we expect the time-derivative of the selection coefficients to be small, that is, $\boldsymbol{s}$ changes slowly over time. By default, we set $\gamma = 1$, which slightly constrains magnitudes of inferred selection coefficients and helps to ensure that the matrix term is invertible. We set $\gamma' = 200$ for time-varying selection coefficients. For constant selection coefficients, we can set a large γ' (e.g., $\gamma' = 10^5$) to obtain a flat result.

With this prior distribution, the overall posterior distribution for the selection coefficients is then given by

$$
\begin{aligned}
P(\boldsymbol{s}|(\boldsymbol{z}(t))_{t_0}^{t_K}) &\propto P((\boldsymbol{z}(t))_{t_0}^{t_K}|\boldsymbol{s}) \cdot P_{\text{prior}}(\boldsymbol{s}(t)) \\
&\propto \exp\left(\boldsymbol{S}_{\text{post}}((\boldsymbol{z}(t))_{t_0}^{t_K}) \right) \,.
\end{aligned}
$$

bioRxiv preprint doi: https://doi.org/10.1101/2025.06.08.658500; this version posted June 9, 2025. The copyright holder for this preprint (which was not certified by peer review) is the author/funder, who has granted bioRxiv a license to display the preprint in perpetuity. It is made available under aCC-BY 4.0 International license.

After applying Bayes's theorem, the action $\boldsymbol{S}((\boldsymbol{z}(t))_{t_0}^{t_K})$ can be modified to $\boldsymbol{S}_{\text{post}}((\boldsymbol{z}(t))_{t_0}^{t_K})$, which can be written as

$$\boldsymbol{S}_{\text{post}}((\boldsymbol{z}(t))_{t_0}^{t_K}) = \boldsymbol{S}((\boldsymbol{z}(t))_{t_0}^{t_K}) + \boldsymbol{S}_{\text{prior}}(\boldsymbol{s},\gamma,\gamma') = \int \boldsymbol{L}(\boldsymbol{z}(t))dt\,. \tag{S7}$$

Here $\boldsymbol{L}$ is the Lagrangian function, with

$$\begin{aligned}\boldsymbol{L}(\boldsymbol{z}(t)) =& \boldsymbol{L}_{\text{path}}(\boldsymbol{z}(t)) + \boldsymbol{L}_{\text{prior}}(\boldsymbol{z}(t)) \\ =& \left\{[\dot{\boldsymbol{z}}^{\mathsf{T}}(t) - \boldsymbol{d}^{\mathsf{T}}(\boldsymbol{z}(t))][\boldsymbol{C}(\boldsymbol{z}(t))]^{-1}[\dot{\boldsymbol{z}}(t) - \boldsymbol{d}(\boldsymbol{z}(t))]\right\} + \left\{\gamma \boldsymbol{s}^{\mathsf{T}}(t)\boldsymbol{s}(t) + \gamma'\dot{\boldsymbol{s}}^{\mathsf{T}}(t)\dot{\boldsymbol{s}}(t)\right\}.\end{aligned} \tag{S8}$$

$\boldsymbol{d}(\boldsymbol{z})$ is the drift vector shown in Eq. S6. In the subsequent discussion, for brevity, we omit the time index t, as it is implicit for most physical quantities. All variables should be understood to be evaluated at t unless explicitly stated otherwise.

Connection between allele level and genotype level

The maximum *a posteriori* selection coefficients can be found by maximizing the action (Eq. S7 and Eq. S8).

$$\hat{\boldsymbol{s}} = \text{arg max}(P(\boldsymbol{s}|(\boldsymbol{z}(t))_{t_0}^{t_K})) = \text{arg max}(\boldsymbol{S}_{\text{post}}(\boldsymbol{z}(t))_{t_0}^{t_K}))$$

This equation contains information at both the genotype level (the genotype fitness gain, $\boldsymbol{h}$ and genotype frequency, $\boldsymbol{z}$) and the allele level (the selection coefficients $\boldsymbol{s}$). To unify the level of selection coefficients, we created a $M \times (\ell + \lambda)$ matrix $\boldsymbol{G}$ that bridges genotype and allele levels, with (a,i)th entries $G_{ai} = g_i^a$ and $(a,\ell+n)$th entries $G_{an} = g_n^a$. This matrix $\boldsymbol{G}$ allows us to express the relationship between genotype fitness gain $\boldsymbol{h}$ and selection coefficients $\boldsymbol{s}$. It also enables us to connect the genotype frequencies $\boldsymbol{z}$ with the allele frequencies $\boldsymbol{x}$.

$$\begin{aligned} h_a &= \sum_i^{\ell} g_i^a s_i + \sum_n^{\lambda} g_n^a s_n, && \rightarrow \boldsymbol{h} = \boldsymbol{G}\cdot\boldsymbol{s}\,; \\ x_i &= \sum_a^{M} g_i^a z_a, x_n = \sum_a^{M} g_n^a z_a, && \rightarrow \boldsymbol{x} = \boldsymbol{G}\cdot\boldsymbol{z}\,. \end{aligned} \tag{S9}$$

Here we denote the frequency of mutant alleles at locus i in the population as x_i, and the frequency of individuals with one or more mutant alleles in binary trait n as x_n. With the connection between $\boldsymbol{h}$ and $\boldsymbol{s}$ established through the matrix $\boldsymbol{G}$, we can rewrite the drift vector in Eq. S7 in terms of the selection coefficients $\boldsymbol{s}$ instead of the genotype fitness gain $\boldsymbol{h}$:

$$\boldsymbol{d}(\boldsymbol{z}) = \boldsymbol{C}(\boldsymbol{z})\cdot\boldsymbol{G}\cdot\boldsymbol{s} + \mu\boldsymbol{E}\cdot\boldsymbol{z} + r(\ell-1)(\boldsymbol{z}-\boldsymbol{\psi})\,, \tag{S10}$$

with $\frac{\partial \boldsymbol{d}(\boldsymbol{z})}{\partial \boldsymbol{s}} = \boldsymbol{C}(\boldsymbol{z})\cdot\boldsymbol{G}$. Here, we need to emphasize that the connection matrix G and the mutation index matrix E are both constant matrices and do not vary with time.

Euler-Lagrange equation for the selection coefficients

Unlike the constant case, we applied the Euler-Lagrange equation $\frac{\partial \boldsymbol{L}}{\partial \boldsymbol{s}} = \frac{d}{dt}(\frac{\partial \boldsymbol{L}}{\partial \dot{\boldsymbol{s}}})$ instead of simple differentiation to maximize the adjusted action (Eq. S7). This approach is necessary due to the time-varying nature of our selection coefficients. To obtain the results more conveniently, we take the transpose of both sides of the equation.

The right side of the equation equals

$$\left[\frac{d}{dt}\left(\frac{\partial \boldsymbol{L}}{\partial \dot{\boldsymbol{s}}}\right)\right]^{\mathsf{T}} = \left[\frac{d}{dt}(2\gamma'\dot{\boldsymbol{s}})\right]^{\mathsf{T}} = 2\gamma'\ddot{\boldsymbol{s}}\,.$$

The left side of the equation is more complex. Given that the covariance matrix $\boldsymbol{C}(\boldsymbol{z})$ is symmetrical, $C^{\mathsf{T}}(\boldsymbol{z})[C(\boldsymbol{z})]^{-1} = C(\boldsymbol{z})[C(\boldsymbol{z})]^{-1} = I$, where I is an identity matrix. LHS can be written as

$$\begin{aligned}\left[\frac{\partial \boldsymbol{L}}{\partial \boldsymbol{s}}\right]^{\mathsf{T}} = \left[\frac{\partial \boldsymbol{L}_{\text{path}}}{\partial \boldsymbol{s}} + \frac{\partial \boldsymbol{L}_{\text{prior}}}{\partial \boldsymbol{s}}\right]^{\mathsf{T}} &= \left(\frac{\partial \boldsymbol{d}}{\partial \boldsymbol{s}}\right)^{\mathsf{T}}\cdot\frac{\partial \boldsymbol{L}}{\partial \boldsymbol{d}} + (2\gamma\boldsymbol{s}^{\mathsf{T}})^{\mathsf{T}} \\ &= (\boldsymbol{C}(\boldsymbol{z})\cdot\boldsymbol{G})^{\mathsf{T}}\cdot\left(-2\left[\boldsymbol{C}(\boldsymbol{z})\right]^{-1}\left[\dot{\boldsymbol{z}} - \boldsymbol{d}(\boldsymbol{z})\right]\right) + 2\gamma\boldsymbol{s} \\ &= -2\boldsymbol{G}^{\mathsf{T}}\cdot[\dot{\boldsymbol{z}} - \boldsymbol{d}(\boldsymbol{z})] + 2\gamma\boldsymbol{s}\,.\end{aligned}$$

By equating these two sides, we obtain:

$$-\boldsymbol{G}^{\mathsf{T}}\dot{\boldsymbol{z}} + \boldsymbol{G}^{\mathsf{T}}\boldsymbol{d}(\boldsymbol{z}) + \gamma\boldsymbol{s} = \gamma'\ddot{\boldsymbol{s}}\,. \tag{S11}$$

bioRxiv preprint doi: https://doi.org/10.1101/2025.06.08.658500; this version posted June 9, 2025. The copyright holder for this preprint (which was not certified by peer review) is the author/funder, who has granted bioRxiv a license to display the preprint in perpetuity. It is made available under aCC-BY 4.0 International license.

The results for the first term can be easily obtained. From equation S9, we can derive that $\dot{\boldsymbol{x}} = \boldsymbol{G}^\top \dot{\boldsymbol{z}}$. The second term is more complicated and requires further detailed analysis. We expand it as follows:

$$\boldsymbol{G}^\top \cdot \boldsymbol{d}(\boldsymbol{z}) = \boldsymbol{G}^\top \cdot \boldsymbol{C}(\boldsymbol{z}) \cdot \boldsymbol{G} \cdot \boldsymbol{s} + \mu \boldsymbol{G}^\top \cdot \boldsymbol{E} \cdot \boldsymbol{z} + r(\ell-1)\boldsymbol{G}^\top \cdot (\boldsymbol{z} - \boldsymbol{\psi}) \,. \tag{S12}$$

Our model consists of three main parts: the covariance matrix, mutation, and recombination. We'll discuss each in turn. For covariance matrix part, we have

$$\boldsymbol{G}^\top \boldsymbol{C}(\boldsymbol{z}) \boldsymbol{G} = \boldsymbol{C}(\boldsymbol{x}) \approx \begin{cases} x_{\boldsymbol{i}}(1 - x_{\boldsymbol{i}}) & \boldsymbol{i} = \boldsymbol{j}\,, \\ x_{\boldsymbol{ij}} - x_{\boldsymbol{i}} x_{\boldsymbol{j}} & \boldsymbol{i} \neq \boldsymbol{j}\,, \end{cases} \tag{S13}$$

Here, $\boldsymbol{i}, \boldsymbol{j}$ is a generic expression for locus i and binary trait n. We typically think of these binary traits as special virtual loci and use the generic index $\boldsymbol{i}, \boldsymbol{j}$. It's important to note that this index is distinct from the index of individual loci, which we denote as i, j.

Next, we examine the mutation term, which requires separate analysis for individual loci and binary traits. For the locus part:

$$\begin{aligned} (\boldsymbol{G}^\top \cdot \boldsymbol{E} \cdot \boldsymbol{z})_i &= \sum_b^M \left(\sum_a^M g_i^a \cdot E_{ab} \right) z_b \\ &= \sum_b^M \sum_{a|d(a,b)=1}^M g_i^a z_b - \sum_b^M g_i^b \ell z_b \end{aligned}$$

We can compute that $\sum_b^M g_i^b \ell z_b = \ell x_i$. The first part requires a more detailed discussion of different scenarios. Since genotypes a and b differ by a single mutation, there are two scenarios:

1. $g_i^a \neq g_i^b$: They differ at locus i, where each b only has one a satisfying the condition. In this scenario, $g_i^a = 1 - g_i^b$.
2. $g_i^a = g_i^b$: They differ at a locus other than i, where each b has $(\ell - 1)$ corresponding a. This leads to:

$$\begin{aligned} \sum_b^M \sum_{a|d(a,b)=1}^M g_i^a z_b &= \sum_b^M \sum_{g_i^a \neq g_i^b}^M (1 - g_i^b) z_b + \sum_b^M \sum_{g_i^a = g_i^b}^M g_i^b z_b \\ &= \sum_b^M (1 - g_i^b) z_b + (\ell - 1) \sum_b^M g_i^b z_b \\ &= (1 - x_i) + (\ell - 1) x_i \,. \end{aligned}$$

Thus, for locus part, the mutation term can be represented as

$$\begin{aligned} (\boldsymbol{G}^\top \cdot \boldsymbol{E} \cdot \boldsymbol{z})_i &= \sum_b^M \sum_{a|d(a,b)=1}^M g_i^a z_b - \sum_b^M g_i^b \ell z_b \\ &= (1 - x_i) + (\ell - 1) x_i - \ell x_i \\ &= 1 - 2x_i \,. \end{aligned} \tag{S14}$$

Using a similar method of case-by-case analysis, we can similarly get the conclude for binary trait part. For this part, given that genotype a and b differ by only one mutation, there are three scenarios:

1. $g_n^a = 0$: This then gives 0 directly regardless of the value of g_n^b.
2. $g_n^a = 1$ while $g_n^b = 0$: For the binary trait n, genotype b contains no mutation while genotype a has only one single mutation, where each genotype b corresponds to $l_n = \sum_{i \in n}$ types of genotype a. This yields $l_n * \sum_b^M (1 - g_n^b) z_b = \sum_{i \in n} (1 - x_n)$.
3. $g_n^a = g_n^b = 1$: Both genotypes a and b have at least one mutation in binary trait n, which is a little complicated. We distinguish two additional cases based on the mutation number for b in binary trait n:
   (a) Single mutation in b: The $a - b$ difference cannot be at b's mutation locus. Each b corresponds to $(\ell - 1)$ genotypes a. The probability for this case is $P_a = \sum_{i \in n} y_n^i / x_n$, where y_n^i means the frequency of genotypes that contain only one mutation in trait group n, and the mutation is in locus i.

bioRxiv preprint doi: https://doi.org/10.1101/2025.06.08.658500; this version posted June 9, 2025. The copyright holder for this preprint (which was not certified by peer review) is the author/funder, who has granted bioRxiv a license to display the preprint in perpetuity. It is made available under aCC-BY 4.0 International license.

(b) Multiple mutations in b: The $a-b$ mutational difference can be at any position. Each b corresponds to ℓ genotypes a. Thus, the results for this part are equal to: $P_a(\ell-1)\sum_b^M g_n^b z_b + (1-P_a)\ell\sum_b^M g_n^b z_b = lx_n - \sum_{i\in n} y_n^i$.

Given the above, for binary trait part, the mutation term can be represented as

$$
\begin{aligned}
(\boldsymbol{G}^\intercal \cdot \boldsymbol{E}\cdot \boldsymbol{z})_n &= \sum_b^M \sum_{a|d(a,b)=1}^M g_n^a z_b - \sum_b^M g_n^b \ell z_b \\
&= \sum_{i\in n}(1-x_n) + lx_n - \sum_{i\in n} y_n^i - \ell x_n \\
&= \sum_{i\in n}\left((1-x_n) - y_n^i\right) .
\end{aligned}
$$

Interestingly, although the equations for individual loci and binary traits appear very different, we can discover that if we consider a single locus as a special binary trait, then the binary trait equation can be simplified to the locus equation. This serves as a verification of the correctness of our derivation for the trait part.

The recombination part is similar to the mutation part in its complexity. We begin with the locus part:

$$
\begin{aligned}
((\ell-1)\boldsymbol{G}^\intercal \cdot (\boldsymbol{z}-\boldsymbol{\psi}))_i &= (\ell-1)\sum_a^M g_i^a z_a - \sum_k^{l-1}\sum_a^M g_i^a \psi_a \\
&= (\ell-1)x_i - \sum_k^{l-1}\sum_a^M g_i^a \psi_a .
\end{aligned}
$$

Here, let k index the $\ell-1$ possible breakpoints for a sequence of length ℓ. To clarify the second term, we replace $\ell-1$ with its expanded form, the sum over all possible breakpoints.

Before discussing the different cases for the second term, we first define a new quantity θ_i^{cd}

$$
\theta_i^{cd} = \sum_a^M g_i^a R_{a,cd}
$$

to represent the probability that the random recombination of any two individuals within the population results in an offspring with mutation at locus i. Here $R_{a,cd}$ is described in equation S3. Thus,

$$
\sum_a^M g_i^a \psi_a = \sum_a^M g_i^a \sum_c^M \sum_d^M R_{a,cd} z_c z_d = \sum_c^M \sum_d^M \theta_i^{cd} z_c z_d . \qquad \textbf{(S15)}
$$

Based on the mutation status for genotype c and d at locus i (values of g_i^c and g_i^d), we have three scenarios:

1. No mutations ($g_i^c = g_i^d = 0$): the offspring produced by recombination cannot have a mutation at i, so $\theta_i^{cd} = 0$.
2. Both mutated ($g_i^c = g_i^d = 1$): Offspring will have a mutation, $\theta_i^{cd} = 1$.
3. Half mutated ($g_i^c = 1, g_i^d = 0$ or $g_i^c = 0, g_i^d = 1$): Half of offspring will have a mutation,$\theta_i^{cd} = 0.5$.

Based on this, Eq. S15 can be represented as

$$
\begin{aligned}
\sum_a^M g_i^a \psi_a =& \sum_{c,d}^M \left((1-g_i^c)(1-g_i^d)\theta_i^{cd} + g_i^c g_i^d \theta_i^{cd} + g_i^c(1-g_d^d)\theta_i^{cd} + (1-g_i^c)g_i^d\theta_i^{cd}\right) z_c z_d \\
=& \sum_{c,d}^M \left(0\cdot(1-g_i^c)(1-g_i^d) + 1\cdot g_i^c g_i^d + \frac{1}{2}g_i^c(1-g_d^d) + \frac{1}{2}(1-g_i^c)g_i^d\right) z_c z_d \\
=& \sum_c^M g_i^c z_c \sum_d^M g_i^d z_d + \frac{1}{2}\sum_c^M g_i^c z_c \sum_d^M (1-g_i^d) z_d + \frac{1}{2}\sum_d^M g_i^d z_d \sum_c^M (1-g_i^c) z_c \\
=& x_i^2 + x_i(1-x_i) \\
=& x_i .
\end{aligned}
$$

bioRxiv preprint doi: https://doi.org/10.1101/2025.06.08.658500; this version posted June 9, 2025. The copyright holder for this preprint (which was not certified by peer review) is the author/funder, who has granted bioRxiv a license to display the preprint in perpetuity. It is made available under aCC-BY 4.0 International license.

This leads to the conclusion that

$$((\ell-1)\boldsymbol{G}^{\mathsf{T}}\cdot(\boldsymbol{z}-\boldsymbol{\psi}))_i = (\ell-1)x_i - \sum_{k}^{l-1} x_i = 0\,.$$

Now consider the binary trait part.

$$\begin{aligned}((\ell-1)\boldsymbol{G}^{\mathsf{T}}\cdot(\boldsymbol{z}-\boldsymbol{\psi}))_n &= (\ell-1)\sum_{a}^{M} g_n^a z_a - \sum_{k}^{l-1}\sum_{a}^{M} g_n^a \psi_a \\ &= (\ell-1)x_n - \sum_{k\in n}\sum_{a}^{M} g_n^a \psi_a\,.\end{aligned}$$

Similarly, we use θ_n^{cd} to represent the probability that recombination can result in an offspring with any mutation in binary trait n. Since a breakpoint can only alter the binary trait's state if it falls within that trait, we restrict the breakpoint range to the binary-trait region rather than the entire sequence - using $\sum_{k\in n}$ instead of $\sum_{k}^{\ell-1}$. We again divide it into three scenarios:

1. No mutations ($g_n^c = g_n^d = 0$): The offspring produced by recombination cannot have any mutation at n, so $\theta_n^{cd} = 0$.

2. Both mutated ($g_n^c = g_n^d = 1$): In most cases, the offspring will have mutations. However, a special case exists where half the offspring lack mutations. This occurs when, within a binary trait n, c has mutations only before break point k, and d only after k. Imagine two parental sequences, c and d, recombining at break point k. Focusing only on mutations within the binary-trait region, sequence c carries mutations exclusively upstream of k, while sequence d carries mutations exclusively downstream. Denoting mutated segments with c tilde ($\sim$), we can write their sequences as: $g^c = (\tilde{g}_1^c,\ldots,\tilde{g}_k^c,g_{k+1}^c,\ldots,g_\ell^c)$ and $g^d = (g_1^d,\ldots,g_k^d,\tilde{g}_{k+1}^d,\ldots,\tilde{g}_\ell^d)$. Then, the sequences for children a and b are $g^a = (\tilde{g}_1^c,\ldots,\tilde{g}_k^c,\tilde{g}_{k+1}^d,\ldots,\tilde{g}_\ell^d)$ and $g^b = (g_1^d,\ldots,g_k^d,g_{k+1}^c,\ldots,g_\ell^c)$. Here, we observe that although both c and d carry mutations ($\sim$), child a, produced by recombining the non-mutated segments of $a1$ and $b1$, contains no mutations. The probability of the special case can be calculated as:

$$P_2 = \frac{P_{M,W}^{k,n}}{x_n}\frac{P_{W,M}^{k,n}}{x_n} + \frac{P_{W,M}^{k,n}}{x_n}\frac{P_{M,W}^{k,n}}{x_n} = \frac{2P_{M,W}^{k,n}P_{W,M}^{k,n}}{x_n^2}$$

   where $P_{M,W}^{k,n}$ represents the frequency of sequences that have at least one mutant allele in the binary trait n before break point k, and all WT alleles in the same binary trait after k. With this probability, we can get θ_n^{cd} in this case:

$$\theta_n^{cd} = (1-P_2)\times 1 + P_2/2 = 1 - P_{M,W}^{k,n}P_{W,M}^{k,n}/x_n^2\,.$$

3. Half mutated ($g_n^c = 1, g_n^d = 0$ or $g_n^c = 0, g_i^d = 1$): In most cases, half of the offspring will have mutations. However, a special case exists where all offspring have mutations. This occurs if the mutant sequence has mutations both before and after breakpoint k. (Parents: $g^c = (\tilde{g}_1^c,\ldots,\tilde{g}_k^c,\tilde{g}_{k+1}^c,\ldots,\tilde{g}_\ell^c)$ and $g^d = (g_1^d,\ldots,g_k^d,g_{k+1}^d,\ldots,g_\ell^d)$. Children: $g^a = (\tilde{g}_1^c,\ldots,\tilde{g}_k^c,g_{k+1}^d,\ldots,g_\ell^d)$ and $g^b = (g_1^d,\ldots,g_k^d,\tilde{g}_{k+1}^c,\ldots,\tilde{g}_\ell^c)$.) The probability can be calculated similarly. Thus, in this scenario,

$$\begin{aligned}P_3 &= \frac{P_{M,M}^{k,n}}{x_n}\frac{P_{W,W}^{k,n}}{1-x_n} \\ \theta_n^{cd} &= (1-P_3)/2 + P_3\times 1 = \frac{1}{2}\left(1+\frac{P_{M,M}^{k,n}P_{W,W}^{k,n}}{x_n(1-x_n)}\right)\end{aligned}$$

   Thus we have

$$\begin{aligned}\sum_{k\in n} g_n^a\psi_a &= \sum_{k\in n}\sum_{c,d}^{M}\left((1-g_n^c)(1-g_n^d)\theta_n^{cd} + g_n^c g_n^d\theta_n^{cd} + g_n^c(1-g_d^d)\theta_n^{cd} + (1-g_n^c)g_n^d\theta_n^{cd}\right)z_c z_d \\ &= \sum_{k\in n}\sum_{c,d}^{M}\left[\left(1-P_{M,W}^{k,n}P_{W,M}^{k,n}/x_n^2\right)\cdot g_n^c g_n^d + 2\cdot\frac{1}{2}\left(1+\frac{P_{M,M}^{k,n}P_{W,W}^{k,n}}{x_n(1-x_n)}\right)g_n^c(1-g_d^d)\right]z_c z_d \\ &= \sum_{k\in n}(x^2 + x_n(1-x_n)) + \sum_{k\in n}(P_{M,W}^{k,n}P_{W,M}^{k,n} - P_{M,M}^{k,n}P_{W,W}^{k,n}) \\ &= (\ell-1)x_n + \sum_{k\in n}(P_{M,W}^{k,n}P_{W,M}^{k,n} - P_{M,M}^{k,n}P_{W,W}^{k,n})\,.\end{aligned}$$

bioRxiv preprint doi: https://doi.org/10.1101/2025.06.08.658500; this version posted June 9, 2025. The copyright holder for this preprint (which was not certified by peer review) is the author/funder, who has granted bioRxiv a license to display the preprint in perpetuity. It is made available under aCC-BY 4.0 International license.

Considering all these scenarios, we can derive the final equation for the binary trait part:

$$((\ell-1)\boldsymbol{G}^{\intercal}\cdot(\boldsymbol{z}-\boldsymbol{\psi}))_n=\sum_{k\in n}(P^{k,n}_{M,M}P^{k,n}_{W,W}-P^{k,n}_{M,W}P^{k,n}_{W,M})\,.$$

Let us now summarize the mutation and recombination terms here:

$$\mu_{fl}=\mu\boldsymbol{G}^{\intercal}\cdot\boldsymbol{E}\cdot\boldsymbol{z}=\begin{cases}\mu(1-2x_i) & \text{Normal locus}\,,\\ \mu\sum_{i\in n}\left((1-x_n)-y_n^i\right) & \text{Binary trait}\,.\end{cases} \tag{S16}$$

$$R_{fl}=r(\ell-1)\left(\boldsymbol{G}^{\intercal}\cdot(\boldsymbol{z}-\boldsymbol{\psi})\right)=\begin{cases}0 & \text{Normal locus}\,,\\ r\sum_{k\in n}(P^{k,n}_{M,M}P^{k,n}_{W,W}-P^{k,n}_{M,W}P^{k,n}_{W,M}) & \text{Binary trait}\,.\end{cases} \tag{S17}$$

Derivation of the differential equation

Building upon our previous derivations (Eqs. S11, S12, S13, S16, S17), we can express the equation in vector form:

$$\begin{aligned}\gamma'\ddot{\boldsymbol{s}}=&\left[\boldsymbol{C}(\boldsymbol{x})+\gamma\boldsymbol{I}\right]\boldsymbol{s}-\dot{\boldsymbol{x}}+\boldsymbol{F}(\boldsymbol{x})+\boldsymbol{R}(\boldsymbol{x})\,;\\ F_i(x)=&\mu(1-2x_i)\,,\\ F_n(x)=&\mu\sum_{i\in n}(1-x_n-y_n^i)\,;\\ R_i(x)=&0\,,\\ R_n(x)=&r\left(\sum_{k\in n}P^{k,n}_{W,W}P^{k,n}_{M,M}-\sum_{k\in n}P^{k,n}_{M,W}P^{k,n}_{M,W}\right)\,.\end{aligned}$$

In this equation, $\boldsymbol{I}$ is the identity matrix, $\boldsymbol{C}(\boldsymbol{x})$ denotes the allele/trait frequency covariance matrix, accounting for evolution speed correlations between mutations/traits, $\dot{\boldsymbol{x}}$ signifies the change in allele/trait frequencies at generation t and $\boldsymbol{F}(\boldsymbol{x})$ and $\boldsymbol{R}(\boldsymbol{x})$ quantify the flux in allele/trait frequencies due to mutation and recombination separately.

Unlike the constant case, the best fit (maximum a posteriori) selection coefficients (Eq. S18) cannot be expressed analytically. This differential equation is second-order with time-varying coefficients (C, F, R) that lack algebraic expressions, precluding an analytical solution. To solve this differential equation, boundary conditions are necessary. However, there are no natural boundary conditions to impose on the selection coefficients $\boldsymbol{s}$, and their values at the endpoints are unknown. This scenario leads to free boundary conditions, resulting in the so-called transversality conditions $\frac{\partial L}{\partial \dot{q}}\big|_{t=0}=0$ and $\frac{\partial L}{\partial \dot{q}}\big|_{t=T}=0$, where L represents the Lagrangian. In our case, these conditions simplify to $\dot{\boldsymbol{s}}(t_0)=0$ and $\dot{\boldsymbol{s}}(t_K)=0$.

Extension to multiple alleles per locus and asymmetric mutation probabilities

To study real sequence data, we can extend the binary allele model presented in the previous sections to allow for multiple alleles per locus. In this section, we will derive results from an allele-level perspective while maintaining consistency with results derived from the genotype-level approach.

We use indices $\alpha,\beta,\ldots$ to represent different alleles, with q denoting the total number of alleles (which could represent, for example, distinct nucleotides or amino acids). To accommodate both multiple-allele states for individual loci and binary states for traits, we adopt the following convention to maintain clarity in our notation: For individual loci, we employ the ordered pair $(i\alpha)$, where i denotes the locus and α specifies the allele at that locus. For binary traits, we retain the notation n for binary traits, which remains unchanged from the binary-allele case, as it naturally exists in only two states (WT and MT). The vector form for mutant allele frequencies at generation t can be written as a vector of length $\ell\ell=\ell q+\lambda$, encompassing all possible allele states at all loci and traits like this $\boldsymbol{x}(t)=(x_{1\alpha}(t),x_{1\beta}(t),\ldots,x_{\ell\alpha}(t),x_{\ell\beta}(t),\ldots,x_{n_1}(t),x_{n_2}(t),$ $\ldots,x_{n_\lambda}(t))$.

The fitness model is expressed as:

$$f_a=1+\sum_{i}^{\ell}\sum_{\alpha}^{q}s_{i,\alpha}g^a_{i,\alpha}+\sum_{n}^{\lambda}s_ng^a_n\,,$$

where $x_{i,\alpha}$ and $s_{i,\alpha}$ represent the frequency and selection coefficient for allele α at locus i respectively, and $g^a_{i,\alpha}$ equals 1 if genotype a has allele α at locus i.

As before, we applied standard methods from statistical physics to convert the Fokker–Planck into a path integral that quantifies the probability density for "path" of mutant allele frequencies $\{\boldsymbol{x}(t_1),\boldsymbol{x}(t_2),...,\boldsymbol{x}(t_K)\}$. The probability for a path

bioRxiv preprint doi: https://doi.org/10.1101/2025.06.08.658500; this version posted June 9, 2025. The copyright holder for this preprint (which was not certified by peer review) is the author/funder, who has granted bioRxiv a license to display the preprint in perpetuity. It is made available under aCC-BY 4.0 International license.

is proportional to the action $\boldsymbol{S}(\boldsymbol{x}(t_k))|_{t_0}^{t_K})$, which is a function of allele frequency $\boldsymbol{x}$ instead of genotype frequency $\boldsymbol{z}$. Before Bayesian inference, the action can be expressed as:

$$\boldsymbol{S}(\boldsymbol{x}(t_k))|_{t_0}^{t_K}) = \sum_{k=0}^{K-1} \frac{1}{\Delta t_k} \left[\boldsymbol{x}^\intercal(t_{k+1}) - \boldsymbol{x}^\intercal(t_k) - \Delta t_k \boldsymbol{d}^\intercal(\boldsymbol{x}(t_k))\right] \left[\boldsymbol{C}(\boldsymbol{x}(t_k))\right]^{-1} \left[\boldsymbol{x}(t_{k+1}) - \boldsymbol{x}(t_k) - \Delta t_k \boldsymbol{d}(\boldsymbol{x}(t_k))\right]$$

$$\Rightarrow \int \left\{[\dot{\boldsymbol{x}}^\intercal(t) - \boldsymbol{d}^\intercal(\boldsymbol{x}(t))][\boldsymbol{C}(\boldsymbol{x}(t))]^{-1}[\dot{\boldsymbol{x}}(t) - \boldsymbol{d}(\boldsymbol{x}(t))]\right\} dt$$

In this formulation, the drift vector $\boldsymbol{d}(\boldsymbol{x}(t))$ and covariance matrix $\boldsymbol{C}(\boldsymbol{x}(t))$ each comprise two distinct components. For simplicity in subsequent discussions, the time parameter t will be implicit unless explicitly required for clarity.

The covariance matrix $\boldsymbol{C}(\boldsymbol{x})$ is defined as an $\ell\ell \times \ell\ell$ matrix. After omitting terms of $\mathcal{O}(1/N^2)$, the covariance matrix can be expressed as:

$$\boldsymbol{C}(\boldsymbol{x}) = \begin{cases} x_{i\alpha}(1 - x_{i\alpha}) & i = j\,, \\ x_{i\alpha,j\beta} - x_{i\alpha}x_{j\beta} & i \neq j, \alpha \neq \beta\,, \\ x_n(1 - x_n) & n = m\,, \\ x_{nm} - x_n x_m & n \neq m\,, \\ x_{i\alpha,n} - x_{i\alpha}x_n & i \neq n\,. \end{cases}$$

which is similar to $\boldsymbol{C}(\boldsymbol{z})$. Here, $x_{i\alpha,j\beta}$ represents the frequency of sequences with allele α and β occurring respectively at loci i and j. $x_{i\alpha,n}$ denotes the frequency of sequences with allele α at locus i and at least one mutation at binary trait n. The covariance between different alleles at the same locus is undefined, as each locus can only exist in one allele state at any given time.

Using previous equations (S6) and neglecting infinitesimals smaller than $\mathcal{O}(1/N)$, the drift vector $d_{\boldsymbol{i}}$ is given by:

$$\begin{aligned} d_{\boldsymbol{i}}(x(t_k)) =& \langle x_{\boldsymbol{i}}(t_{k+1}) - x_{\boldsymbol{i}}(t_k)\rangle \\ =& \sum_a^M g_{\boldsymbol{i}}^a (p_a(z) - z_a) \\ =& \sum_a^M g_{\boldsymbol{i}}^a \left(\sum_{\boldsymbol{j}}^{\ell+\lambda} s_{\boldsymbol{j}} g_{\boldsymbol{j}}^a - \sum_b^M \sum_{\boldsymbol{j}}^{\ell+\lambda} s_{\boldsymbol{j}} g_{\boldsymbol{j}}^b z_b \right) z_a + \sum_a^M g_{\boldsymbol{i}}^a \left[\sum_{b|d(a,b)=1} (\mu_{ab} z_b - \mu_{ba} z_a) \right] + r(\ell - 1) \sum_a^M g_{\boldsymbol{i}}^a \left(z_a(t_k) - \psi_a(t_k)\right) \\ =& \sum_{\boldsymbol{j}}^{\ell+\lambda} \boldsymbol{C}_{\boldsymbol{ij}}(x(t_k)) \boldsymbol{s}_{\boldsymbol{j}} + F_{\boldsymbol{i}}(t_k) + R_{\boldsymbol{i}}(t_k)\,, \end{aligned}$$

with

$$F_{\boldsymbol{i}} = \sum_a^M g_{\boldsymbol{i}}^a \left[\sum_{b|d(a,b)=1} (\mu_{ab} z_b - \mu_{ba} z_a) \right], \qquad R_{\boldsymbol{i}} = r(\ell - 1) \sum_a^M g_{\boldsymbol{i}}^a \left(z_a(t_k) - \psi_a(t_k)\right).$$

Here $\boldsymbol{i}$ is a generic locus, including pair (i, α) for individual loci and simple n for binary traits. This expression generalizes our previous formulations (Eq. S10) from the binary allele to the multiple allele case, incorporating the additional term g. This approach bridges allele-level and genotype-level perspectives. For the mutation term, we employ an asymmetric mutation matrix where the entry μ_{ab} represents the probability per locus per generation of mutation from genotype a to genotype b.

In the following discussion, we forgo a detailed derivation, instead focusing on the result and its physical interpretation. Since the binary trait part is not affected by the transition to the multiple allele case, and the recombination part for individual loci equals 0 (as recombination cannot alter individual allele frequencies), the recombination expression remains the same as before but requires careful consideration of synonymous mutations:

$$\boldsymbol{R}(\boldsymbol{x}) = \begin{cases} 0\,, \\ r\left(\sum_{k \in n} P_{W,W}^{k,n} P_{M,M}^{k,n} - \sum_{k \in n} P_{M,W}^{k,n} P_{M,W}^{k,n} \right). \end{cases}$$

It's crucial to note that synonymous mutant alleles do not contribute to binary traits and should be considered as wild-type alleles when calculating the recombination component for binary traits. The result here quantifies the net increase or decrease in mutant allele or binary trait frequency over time due to recombination.

bioRxiv preprint doi: https://doi.org/10.1101/2025.06.08.658500; this version posted June 9, 2025. The copyright holder for this preprint (which was not certified by peer review) is the author/funder, who has granted bioRxiv a license to display the preprint in perpetuity. It is made available under aCC-BY 4.0 International license.

The mutation component is more complex, requiring careful consideration of transitions between different alleles at the same locus. For an individual locus, the mutation component is expressed as:

$$F_{i,\alpha}(x) = \sum_{\beta\neq\alpha}^{q} \left(\mu_{\beta\alpha} x_{i,\beta} - \mu_{\alpha\beta} x_{i,\alpha}\right).$$

Here $\mu_{\alpha\beta}$ represents the probability per locus per generation of mutation from allele α to allele β. In this formulation, $\mu_{\beta\alpha} x_{i,\beta}$ quantifies the probability of allele β mutating to allele α at locus i. When summed over all possible alleles, this expression yields the net frequency change due to mutation. When $q = 2$ and assuming only one μ, this equation can be simplified to the binary case, given by Eq. S14.

The treatment of binary traits requires special consideration of synonymous mutations. Unlike individual loci, binary traits may have more than one "wild type." We introduce δ to denote nonsynonymous mutant alleles, while ϵ denotes synonymous mutant alleles and wild type alleles. The mutation component for binary traits is expressed as:

$$F_n(x) = \sum_{i\in n}\sum_{\delta}\sum_{\epsilon} \left(\mu_{\epsilon\delta} y_n^{i\epsilon} - \mu_{\delta\epsilon} y_n^{i\delta}\right).$$

Here, $y_n^{i\delta}$ represents the frequency of sequences containing only one nonsynonymous mutation in trait group n, with mutation δ in locus i. This expression accounts for all loci within binary trait n, encompassing all possible nonsynonymous mutant alleles δ and all possible synonymous mutant alleles or wild type alleles ϵ. In the binary case, there is only one nonsynonymous mutation δ, and synonymous mutations ϵ can be seen as wild type. Thus, $y_n^{i\epsilon}$ means the frequency for wild type in the binary trait n, which is $(1 - x_n)$.

Our generalized formulation demonstrates consistency with simpler models through reduction to the binary-allele version under specific conditions. In the absence of synonymous mutant alleles within the binary trait, $y_n^{i\epsilon}$ is the frequency of wild type for binary trait n, equal to $1 - x_n$. When each trait only has a single δ and a single ϵ, our expression simplifies to match the binary-allele formulation. This reduction validates our approach, confirming its ability to encompass both simple and complex scenarios within a unified framework.

Thus, the final equation is

$$\begin{aligned}
\gamma'\ddot{\boldsymbol{s}} &= [\boldsymbol{C}(\boldsymbol{x}) + \gamma \boldsymbol{I}]\,\boldsymbol{s} - \dot{\boldsymbol{x}} + \boldsymbol{F}(\boldsymbol{x}) + \boldsymbol{R}(\boldsymbol{x})\,; \qquad \textbf{(S18)}\\
F_{i,\alpha}(x) &= \sum_{\beta\neq\alpha}^{q} \left(\mu_{\beta\alpha} x_{i,\beta} - \mu_{\alpha\beta} x_{i,\alpha}\right),\\
F_n(x) &= \sum_{i\in n}\sum_{\delta}\sum_{\epsilon} \left(\mu_{\epsilon\delta} y_n^{i,\epsilon} - \mu_{\delta\epsilon} y_n^{i,\delta}\right);\\
R_{i,\alpha}(x) &= 0\,,\\
R_n(x) &= r\left(\sum_{k\in n} P_{W,W}^{k,n} P_{M,M}^{k,n} - \sum_{k\in n} P_{M,W}^{k,n} P_{M,W}^{k,n}\right).
\end{aligned}$$

Domain extension method

To apply the domain extension method to our equation, we first create a new extended domain $\tilde{\Omega}$. We extend the total generation by half in both forward and backward directions. The original time range in Ω spans from t_0 to t_K. In the extended domain $\tilde{\Omega}$, we add a segment of length $\tilde{t}_0 - \tilde{t}_i$ before t_0, and a segment of length $\tilde{t}_e - \tilde{t}_K$ after t_K, each equal to $(t_K - t_0)/2$ (illustrated in **Supplementary Fig. S2**a). Then, we can set our extended transversality conditions:

$$\dot{\boldsymbol{s}}(\tilde{t}_0) = \dot{\boldsymbol{s}}(\tilde{t}_K) = 0\,.$$

The source term in the extended domain $\tilde{\Omega}$ will be zeroed out. In addition, the covariance matrix will also be removed since there are no evolutionary forces outside the original region. Thus, the equation in $\tilde{\Omega}$ is only influenced by regularization force:

$$\gamma'\ddot{\boldsymbol{s}} = \gamma \boldsymbol{I}\boldsymbol{s}$$

To ensure a smooth transition of the solution, we require continuity of the value of the solution and its derivative along the boundary $\partial\Omega$:

$$\begin{aligned}
\boldsymbol{s}(t_0) = \boldsymbol{s}(\tilde{t}_0)\,, \boldsymbol{s}(t_K) = \boldsymbol{s}(\tilde{t}_K)\,;\\
\dot{\boldsymbol{s}}(t_0) = \dot{\boldsymbol{s}}(\tilde{t}_0)\,, \dot{\boldsymbol{s}}(t_K) = \dot{\boldsymbol{s}}(\tilde{t}_K)\,.
\end{aligned}$$

With all these equations and conditions, we can finally optimize the selection coefficients from t_0 to t_K.

bioRxiv preprint doi: https://doi.org/10.1101/2025.06.08.658500; this version posted June 9, 2025. The copyright holder for this preprint (which was not certified by peer review) is the author/funder, who has granted bioRxiv a license to display the preprint in perpetuity. It is made available under aCC-BY 4.0 International license.

Testing performance in simulations

We conducted simulations of the time-varying Wright-Fisher model with discrete generations and binary (mutant/WT) states using Python. Our analysis encompassed two scenarios: a simple case without binary traits and a more complex case incorporating binary traits. For the simple case, we employed a simplified fitness model:

$$f_a(t) = 1 + \sum_{i}^{\ell} s_i(t) g_i^a(t)$$

The complex case involved evolving populations of sequences according to our previously defined fitness equation (S1) over multiple generations.

For both cases, we started with an initial population of 4 random genotypes (each locus having a 20% probability of being mutant type). The value of γ is different from the value we used in constant inference. In constant inference, γ appears with $C_{int} = \sum_{k=0}^{K} \Delta t_k C(t_k)$, the sum of covariance matrix over all generations; while in the time-varying case, γ appears with $C(t_k)$, the covariance matrix at that generation. Terms in C_{int} and $C(t_k)$ can vary over several orders of magnitude, depending on the length of the trajectory. Thus, we need to use a smaller value regularization strength, such as $\gamma = 10^{-3}$, to constrain the magnitude of the inferred time-varying selection coefficients. For the γ' part, as in (**Supplementary Figs. S2** and **S5**), we extended the time range and used a time-varying value. The value we used for the boundary extended time is 4 times larger than the center time, which exponentially decreases to the middle value in a short time (10% of the total generations). For the center value, we set $\gamma' = 200$ for time-dependent selection coefficients and $\gamma' = 10^6$ for time-independent selection coefficients. Parameter values are detailed in **Fig. 2** and **Supplementary Fig. S3** respectively.

Influence of γ' on inference

As discussed in the main text, the time coordinate in (2) practically behaves like a spatial coordinate in the context of inference. Similar to solving the electric field problem in Poisson's equation, we have extended the range of generation t by adding half of the total generations to both the beginning and the end. For this outside area, all terms except for the two regularization terms due to the prior distribution will disappear, since we imagine there is no evolutionary force at those times. The value of γ', which quantifies the expected time scale of environmental fluctuations, can influence the inference results. As described in the main text, $1/(2\gamma')$ is the variance of $\dot{s}(t)$, which means that a higher value of γ' results in a smoother estimated result. At the boundary time points, we tend to use a large γ' to avoid large fluctuations of selection or trait coefficient trajectories, which can change rapidly compared to times in the "bulk", far from t_0 and t_K. Here we allocate 10% of of the total generations for γ' to change its value, which is 100 generations in our simulations.

From repeated simulations (**Supplementary Figs. S2-S5**), we assessed how our method performs for different γ' and could see a trade-off between average value and variance. A smaller γ' means larger fluctuations and a larger variance of the inferred value (**Supplementary Fig. S2a**), while a larger γ' can lead to a smoother inferred results (**Supplementary Fig. S2c**). When we have limited data, we tend to choose a larger γ' at boundaries and that quickly reverts to normal values to prevent significant fluctuations at these points. However, in data sets with multiple replicates, a smaller γ' typically yields an average value closer to the true value, particularly when the true value deviates significantly from 0. This comes at the cost of potentially extreme individual inference results.

We also observe that the root mean square error for the estimated selection coefficient is very large at the end of the trajectory. Even when we set a symmetric boundary condition for inference, the root mean square error is not symmetric. This is because we began with only 4 genotypes and ended with hundreds of genotypes, making the situation at the end of the trajectory much more complicated than the beginning.

HIV-1 data

We obtained HIV-1 sequence data from 13 individuals of the CHAVI 001 and CAPRISA 002 studies in the United States, Malawi, and South Africa from the Los Alamos National Laboratory (LANL) HIV Sequence Database. For each individual, longitudinal HIV-1 half-genome sequences were collected from around the time of peak infection up to several months or years afterward. Donors did not receive antiretroviral drug treatment during this study.

Our data preparation process involved several steps to ensure data quality. We focused on heavily-sequenced half-genome regions, trimming full-length sequences accordingly. To minimize noise, we applied multiple selection criteria: We clipped full-length sequences down to the heavily-sequenced half-genome regions and applied several selection criteria to minimize the influence of noise in the data, including removing the sequences with large numbers of gaps, loci with high gap frequencies ($>= 95\%$ gaps), and time points with very small numbers of sequences (< 4) or large gaps (> 300) in time from the last sample. Following the removal of poorly sampled data points, ambiguous nucleotides were replaced by the most frequently observed nucleotides at the same site within the same individual. The final dataset consisted of $3'$ and $5'$ half-genome sequences of approximately 4,500 base pairs (bp) in length. Our analysis focused on polymorphic sites, defined as locations where more

bioRxiv preprint doi: https://doi.org/10.1101/2025.06.08.658500; this version posted June 9, 2025. The copyright holder for this preprint (which was not certified by peer review) is the author/funder, who has granted bioRxiv a license to display the preprint in perpetuity. It is made available under aCC-BY 4.0 International license.

than one nucleotide (including gaps/deletions) was observed within an individual, typically around 100-900 bp in total. We designated the transmitted/founder (TF) sequence as the "wild-type" sequence for each individual. As noted above, synonymous mutant alleles within binary traits were considered "wild-type" for the purpose of computing the trait frequency.

The locations of CD8^+ T cell epitopes in these sequences were determined both experimentally[60] and computationally[61]. To isolate the fitness effects of escape from individual mutation effects, we focused on escape effects that could be independently inferred from other fitness contributions. This required that the escape trait be neither completely correlated nor anti-correlated with other variants. We accomplished this by reducing the integrated covariance matrix $C_{int} = \sum_t C(t_k)\Delta t$ to its reduced row-echelon form (RREF) and checking the linear dependencies of the rows for epitopes. The epitopes whose corresponding rows of the integrated covariance matrix are linearly independent are denoted as binary traits (ref.[57,62]). These typically include epitopes containing three or more loci with non-synonymous mutations, though two loci may sometimes suffice. "Escape sites" are defined as polymorphic sites whose non-synonymous mutations were observed in the reading frame of an independent CD8^+ T cell epitope. We anticipated that these non-synonymous mutations in escape sites would affect T cell recognition. Sites whose mutations can change the same epitope were considered part of a single "trait group."

To infer selection, we used a mutation rate matrix[63] and a virus-load (VL) dependent recombination rate[64] as input. In our model, we dynamically determined the recombination rate based on the viral load at each time point (a simple linear model with $r = 1.722 \times 10^{-10}\,\text{VL} + 1.39 \times 10^{-5}$), which was measured in past work[60]. For later stages of infection where virus load was not measured, we assumed that VL values remained unchanged from the most recent measurement, consistent with the establishment of a viral set point in chronic infection.

We observed that the SR10 epitope in CH040 exhibited a relatively high escape frequency at the initial time point. To ensure biological plausibility of our results, we introduced a "negative time point" (-7 days) for CH040, roughly representing transmission, where all sequences were set to the TF sequence. This adjustment point can be observed in **Fig. 4b** and **Supplementary Fig. S7a**.

In our analysis, we employed γ to constrain the magnitudes of inferred coefficients, using different values for individual loci and binary traits: $\gamma = 1/\max(t)$ and $\gamma = 10/\max(t)$, respectively. Based on biological expectations, we also implemented asymmetric constraints. Specifically, we strongly expect that CTL escape should be beneficial for the virus, or at the very least, it should not be harmful to viral replication. We emphasize that this does *not* mean that individual escape mutations cannot be deleterious, or even that escape may be a net detriment to viral replication (that is, the benefit of escape may be outweighed by profoundly deleterious escape mutations). Rather, we claim that the property of being unrecognizable to CTLs on its own should not be harmful. Thus, the distribution of escape coefficients should not be symmetric around $s = 0$. Specifically, the variance in the $s < 0$ region should be smaller than that in the $s > 0$ region. Therefore, we applied a substantial penalty to negative escape coefficients by multiplying the corresponding γ values by 100. We also observed that once a mutation reaches fixation ($x = 1$), the γ parameter tends to drive the inferred selection coefficients toward zero due to a lack of evidence for persistent positive selection. To prevent the suppression of selection coefficients at later time points due to regularization effects alone, we implemented a smaller γ (1/10) after a mutation becomes fixed. For γ', we used a constant value ($\gamma' = 10^6$) for time-independent selection coefficients and a time-dependent value (a similar pattern to **Supplementary Fig. S2c** in simulations, with a center value equal to 50, which is smaller than the value in simulations) for time-dependent coefficients, accounting for the sudden frequency changes characteristic of HIV-1 data.

Data and code

Raw data and code used in our analysis are available in the GitHub repository located at `https://github.com/bartonlab/paper-time-varying-selection`. This repository also contains Jupyter notebooks that can be run to reproduce the results presented here.

References

57. Gao, Y. & Barton, J. P. A binary trait model reveals the fitness effects of hiv-1 escape from t cell responses. *Proceedings of the National Academy of Sciences* **122**, e2405379122 (2025).
58. Ewens, W. J. *Mathematical population genetics: theoretical introduction*, vol. 1 (Springer, 2004).
59. Risken, H. & Risken, H. *Fokker-planck equation* (Springer, 1996).
60. Liu, M. K. *et al.* Vertical t cell immunodominance and epitope entropy determine hiv-1 escape. *The Journal of clinical investigation* **123** (2012).
61. Barton, J. P. *et al.* Relative rate and location of intra-host hiv evolution to evade cellular immunity are predictable. *Nature communications* **7**, 11660 (2016).
62. Sohail, M. S., Louie, R. H., Hong, Z., Barton, J. P. & McKay, M. R. Inferring epistasis from genetic time-series data. *Molecular biology and evolution* **39**, msac199 (2022).
63. Zanini, F., Puller, V., Brodin, J., Albert, J. & Neher, R. A. In vivo mutation rates and the landscape of fitness costs of hiv-1. *Virus evolution* **3**, vex003 (2017).
64. Romero, E. V. & Feder, A. F. Elevated hiv viral load is associated with higher recombination rate in vivo. *Molecular Biology and Evolution* **41**, msad260 (2024).

bioRxiv preprint doi: https://doi.org/10.1101/2025.06.08.658500; this version posted June 9, 2025. The copyright holder for this preprint (which was not certified by peer review) is the author/funder, who has granted bioRxiv a license to display the preprint in perpetuity. It is made available under aCC-BY 4.0 International license.

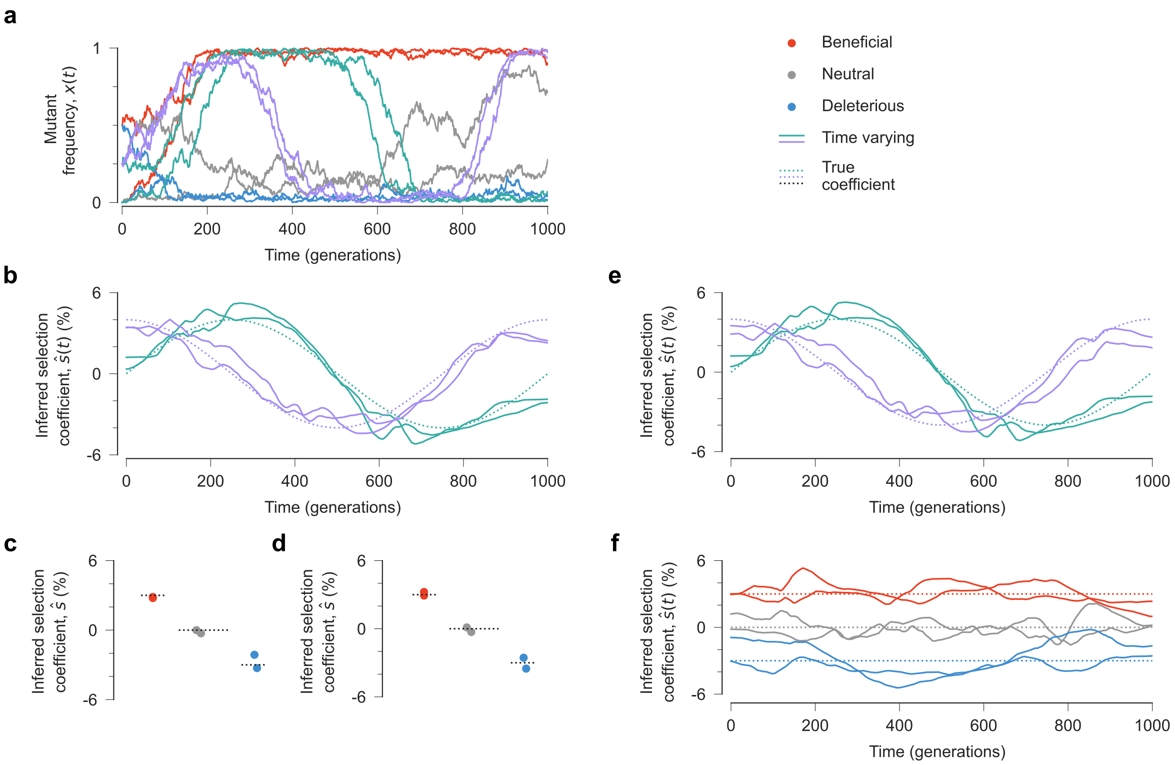

Supplementary Fig. S1. Robustness to model misspecification. **a**, Simulated mutant allele frequency trajectories. Time-varying selection coefficients (**b**) and constant ones (**c**) inferred with prior knowledge of the constant sites. To test the robustness of our approach to model misspecification, we also tested a scenario where all selection coefficients were assumed to be time-varying, including the constant ones. In that case we obtained time-varying coefficients (**e**) that were very similar to the previous ones. Inferred selection for the constant coefficients (**f**) fluctuated around the true, underlying constant values. To help visualize the long-term average, we plotted the mean of these inferred selection coefficients over time (**d**) versus their true, constant values. Simulation parameters: $\ell = 10$ loci with two alleles at each locus, two beneficial mutants with $s = 0.03$, two neutral mutants with $s = 0$ and two deleterious mutants with $s = -0.03$. Additionally, four mutations have fitness effects that vary over time, with two following a sinusoidal pattern (green) and the others a cosine pattern (purple). The mutation probability per site per generation is $\mu = 1 \times 10^{-3}$, the recombination probability per site per generation is $r = 1 \times 10^{-3}$, and the population size is $N = 10^3$. The initial population was randomly generated and evolved over $T = 1000$ generations.

bioRxiv preprint doi: https://doi.org/10.1101/2025.06.08.658500; this version posted June 9, 2025. The copyright holder for this preprint (which was not certified by peer review) is the author/funder, who has granted bioRxiv a license to display the preprint in perpetuity. It is made available under aCC-BY 4.0 International license.

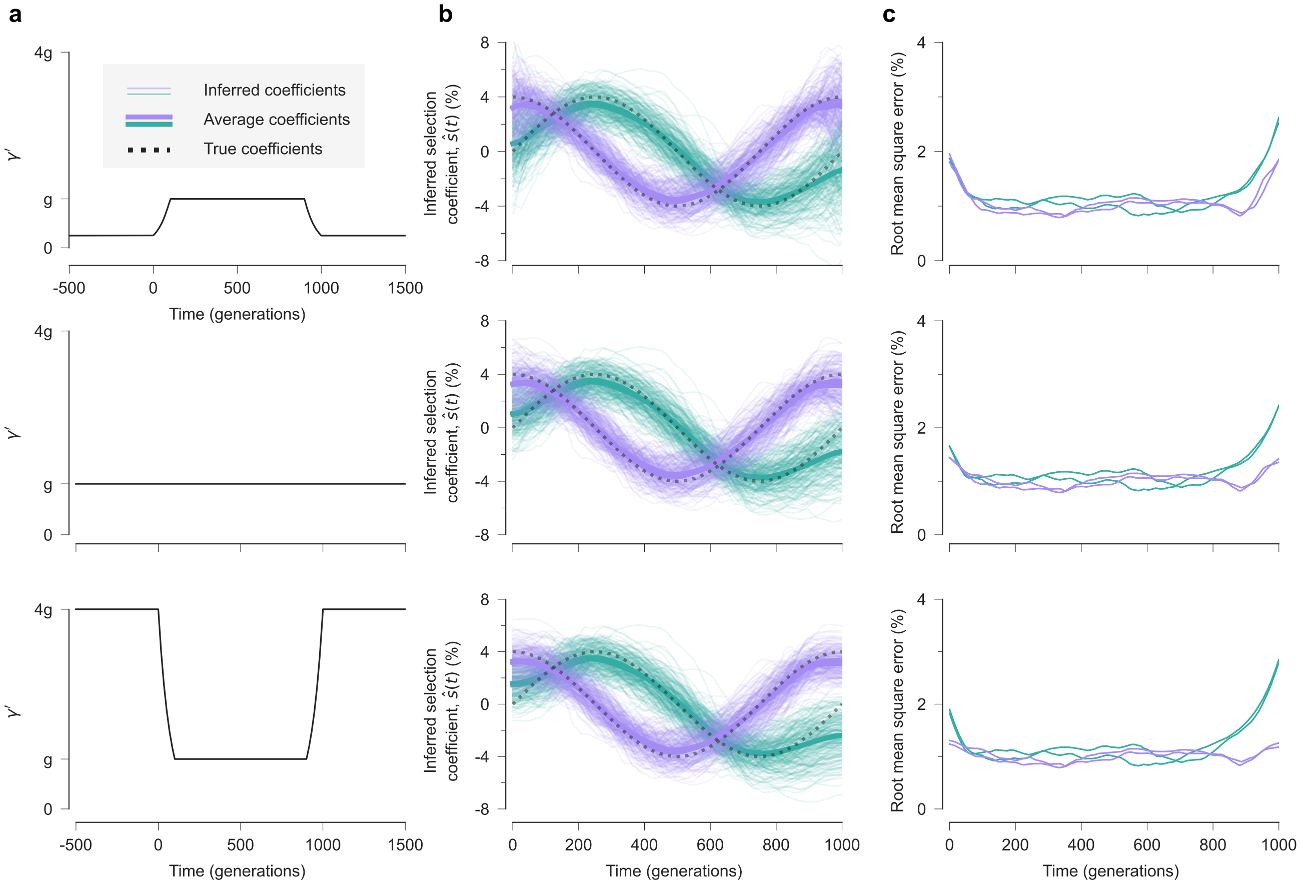

Supplementary Fig. S2. Effects of different γ' profiles on inference near the boundary. In real data and simulations, the data that we use for inference is bounded between some initial time t_0 and final time t_K (here zero and 1000, respectively). Selection coefficients near t_0 and t_K are less well-constrained than those in the "bulk" far from the time boundaries because we receive no information from times before t_0 or after t_K. Here, we examined how changes in γ' in the bulk and beyond the boundaries affect our inference. Column **a** shows the change of γ' across time. Time before 0 and after 1000 are the extended time. Column **b** displays the inferred time-varying selection coefficients and their averages across 100 independent simulations with identical underlying fitness parameters. Column **c** shows the root mean square error of inferred values across 100 simulations as a function of time. The inferred coefficients closely match the true values in the central time region across all cases. However, when γ' takes small values near the extended boundaries, the inferred coefficients exhibit significant fluctuations in these regions, with a higher RMSE. Increasing γ' near the boundaries suppresses fluctuations, which can sometimes decrease inference errors. Simulation parameters: $\ell = 10$ loci with two alleles at each locus (mutant and wild-type, WT), two beneficial mutants with $s = 0.02$ (red), two neutral mutants with $s = 0$ (grey), and two deleterious mutants with $s = -0.02$ (blue). We consider four mutations with time-varying fitness effects, with two following a sinusoidal pattern (green) and the others a cosine pattern (purple). The mutation probability per site per generation is $\mu = 1 \times 10^{-3}$, the recombination probability per site per generation is $r = 1 \times 10^{-3}$, and the population size is $N = 10^3$. The initial population was randomly generated and evolved over $T = 1000$ generations, with each locus having a 20% probability of being mutant type.

bioRxiv preprint doi: https://doi.org/10.1101/2025.06.08.658500; this version posted June 9, 2025. The copyright holder for this preprint (which was not certified by peer review) is the author/funder, who has granted bioRxiv a license to display the preprint in perpetuity. It is made available under aCC-BY 4.0 International license.

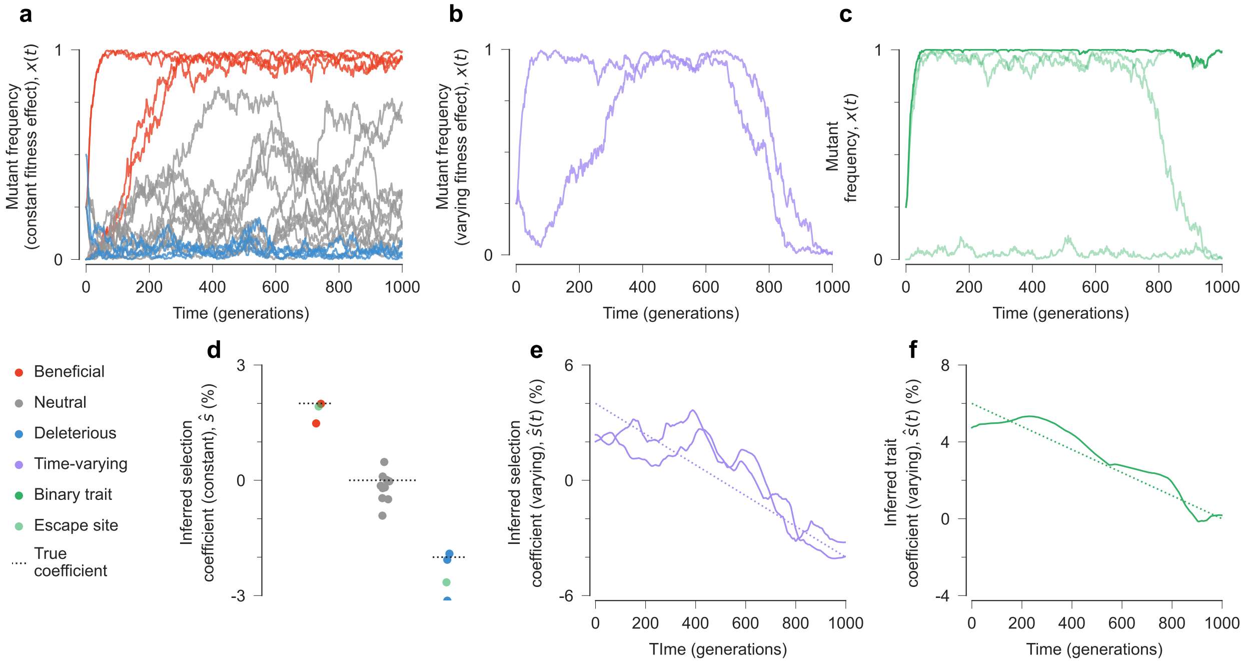

Supplementary Fig. S3. Inferring selection on binary traits with time-varying fitness effects from temporal genetic data. **a**, Frequency trajectories for alleles with constant fitness effects. **b**, Frequency trajectories for alleles with time-varying fitness effects. **c**, Trait frequencies and their contributing individual allele frequencies in the same simulation. The inferred constant selection coefficients (**d**), time-varying selection coefficients(**e**), and time-varying trait coefficients (**f**) are close to their true values. Simulation parameters: $\ell = 20$ loci with two alleles at each locus (mutant and wild-type, WT) including several mutations with constant fitness effects (beneficial mutants with $s = 0.02$ (red), neutral mutants with $s = 0$ (gray), deleterious mutants with $s = -0.02$ (blue) and mutants contributing to the binary trait (green)) and two mutations with time-varying fitness effects. Green mutations have two distinct effects on fitness: one through their individual selection coefficients (**d**) and one through their contribution to the time-varying trait effect (**e**), analogous to immune escape in the context of HIV-1. The mutation probability per site per generation is $\mu = 1 \times 10^{-3}$, the recombination probability per site per generation is $r = 1 \times 10^{-3}$, and the population size is $N = 10^3$. The initial population was randomly generated and evolved over $T = 1000$ generations, with each locus having a 20% probability of being mutant type.

bioRxiv preprint doi: https://doi.org/10.1101/2025.06.08.658500; this version posted June 9, 2025. The copyright holder for this preprint (which was not certified by peer review) is the author/funder, who has granted bioRxiv a license to display the preprint in perpetuity. It is made available under aCC-BY 4.0 International license.

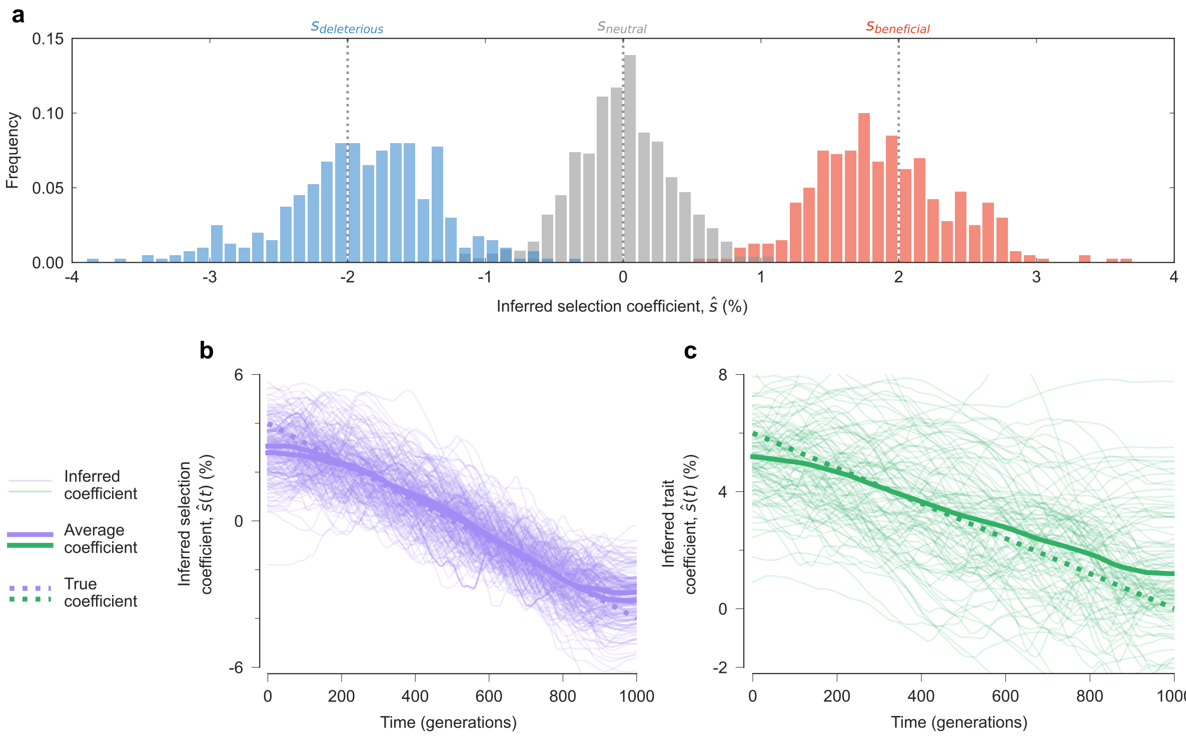

Supplementary Fig. S4. Consistency of inference across multiple replicate simulations including time-varying fitness effects of individual mutations and binary traits. Distribution of **a** inferred constant selection coefficient estimated across 100 replicate simulations, using the same initial parameters. **b** and **c** show the inferred time-varying selection coefficients for individual mutant alleles and the binary trait, respectively. Simulation parameters: $\ell = 20$ loci with two alleles at each locus (mutant and wild-type, WT) including several constant mutations (beneficial mutants with $s = 0.02$ (red), neutral mutants with $s = 0$ (gray), deleterious mutants with $s = -0.02$ (blue)) and two time-varying mutations (purple)). Sites contributing to binary traits (green) in these 100 simulations were randomly selected from sites with constant selection coefficients. The mutation probability per site per generation is $\mu = 1 \times 10^{-3}$, the recombination probability per site per generation is $r = 1 \times 10^{-3}$, and the population size is $N = 10^3$. The initial population was randomly generated and evolved over $T = 1000$ generations, with each locus having a 20% probability of being mutant type.

bioRxiv preprint doi: https://doi.org/10.1101/2025.06.08.658500; this version posted June 9, 2025. The copyright holder for this preprint (which was not certified by peer review) is the author/funder, who has granted bioRxiv a license to display the preprint in perpetuity. It is made available under aCC-BY 4.0 International license.

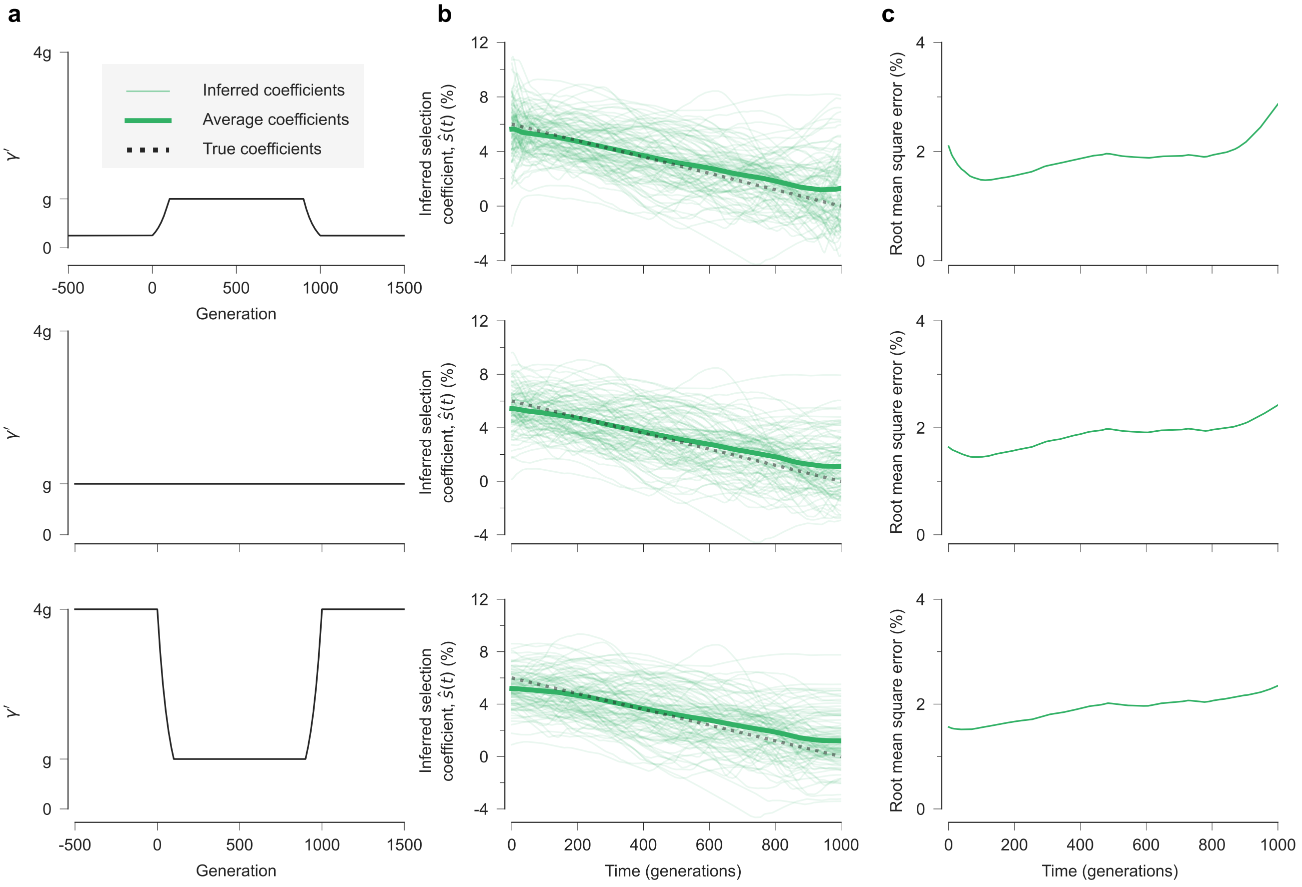

Supplementary Fig. S5. Effects of different γ' profiles for inference of selection on binary traits near the time boundaries. This figure is analogous to **Supplementary Fig. S2**, but here we explore the effect on trait inference. **a**, Profiles of γ' over time. Times before 0 and after 1000 are in the extended domain, beyond the bounds of the simulation. **b**, Inferred time-varying trait coefficients and their average values across 100 independent simulations using identical initial parameters. Here we used the same simulation parameters as in **Supplementary Fig. S3**. **c**, Root mean square error of inferred values across 100 simulations as a function of time. The inferred coefficients closely match the true values in the central time region across all cases. However, when γ' uses small values near the extended boundaries, the inferred coefficients exhibit significant fluctuations in these regions, with a higher RMSE. Increasing γ' around the boundaries suppresses fluctuations due to the lack of constraints in the extended domain. Simulation parameters: $\ell = 20$ loci with two alleles at each locus (mutant and wild-type, WT) including several constant mutations (beneficial mutants with $s = 0.02$, neutral mutants with $s = 0$, deleterious mutants with $s = -0.02$), three random mutants that contribute to the binary trait, and two mutations with time-varying fitness effects. The mutation probability per site per generation is $\mu = 1 \times 10^{-3}$, the recombination probability per site per generation is $r = 1 \times 10^{-3}$, and the population size is $N = 10^3$. The initial population was randomly generated, with each locus having a 20% probability of being mutant type, and evolved over $T = 1000$ generations.

bioRxiv preprint doi: https://doi.org/10.1101/2025.06.08.658500; this version posted June 9, 2025. The copyright holder for this preprint (which was not certified by peer review) is the author/funder, who has granted bioRxiv a license to display the preprint in perpetuity. It is made available under aCC-BY 4.0 International license.

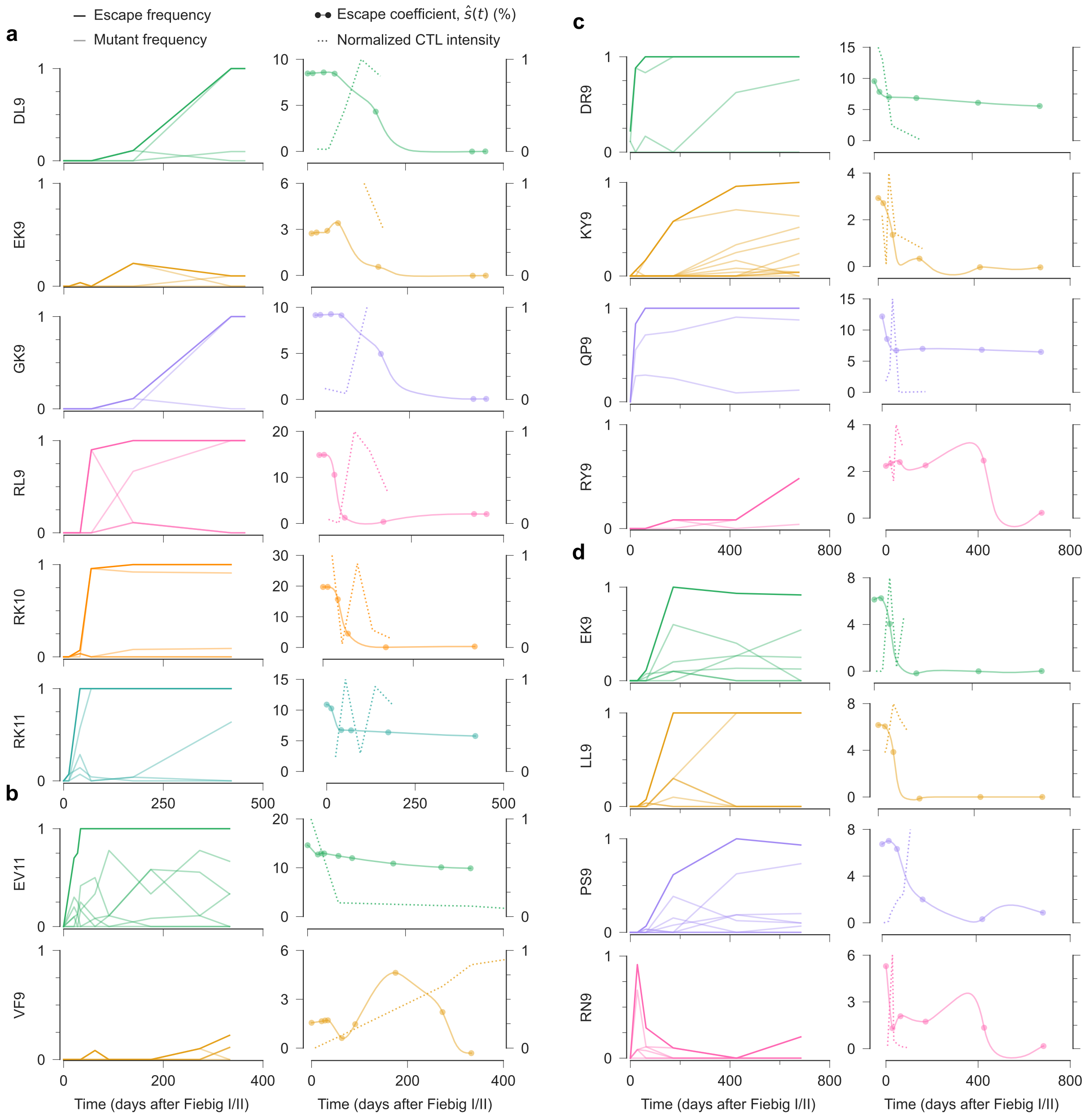

Supplementary Fig. S6. Comparing time-varying selection for CTL escape and experimentally measured CTL intensity. For each CTL epitope with measured intensity data[60], we show the frequency of individual escape mutations and the overall fraction of viruses with one or more escape mutations (left column) along with the inferred escape coefficient and normalized CTL intensity over time (right column). This figure shows CTL epitopes for donors (**a**) CH470, (**b**) CH131, (**c**) CH042, and (**d**) CH256.

bioRxiv preprint doi: https://doi.org/10.1101/2025.06.08.658500; this version posted June 9, 2025. The copyright holder for this preprint (which was not certified by peer review) is the author/funder, who has granted bioRxiv a license to display the preprint in perpetuity. It is made available under aCC-BY 4.0 International license.

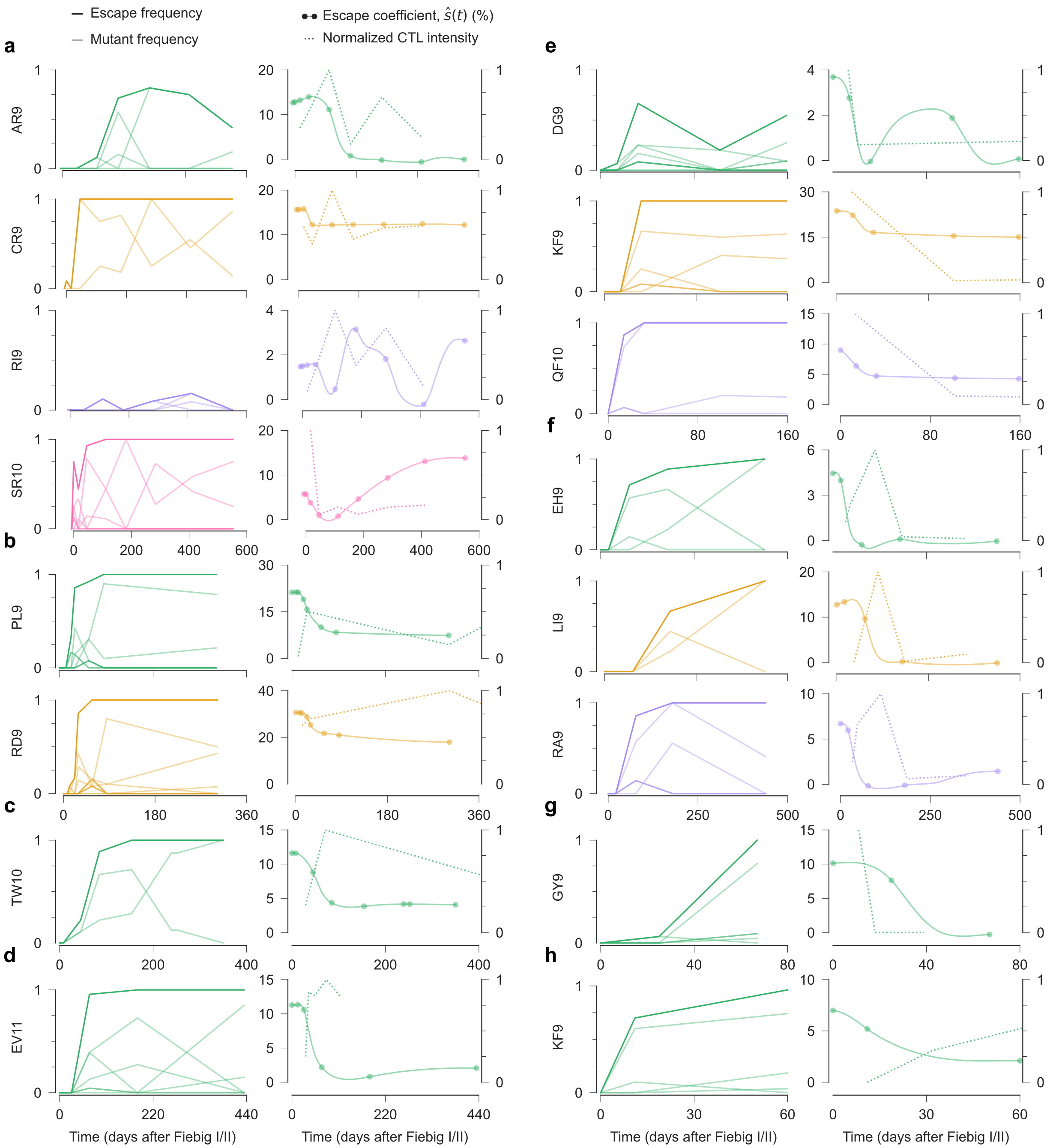

Supplementary Fig. S7. Comparing time-varying selection for CTL escape and experimentally measured CTL intensity. For each CTL epitope with measured intensity data[60], we show the frequency of individual escape mutations and the overall fraction of viruses with one or more escape mutations (left column) along with the inferred escape coefficient and normalized CTL intensity over time (right column). This figure shows CTL epitopes for donors (**a**) CH040, (**b**) CH159, (**c**) CH058, (**d**) CH164, (**e**) CH077, (**f**) CH162, (**g**) CH185, and (**h**) CH198.

bioRxiv preprint doi: https://doi.org/10.1101/2025.06.08.658500; this version posted June 9, 2025. The copyright holder for this preprint (which was not certified by peer review) is the author/funder, who has granted bioRxiv a license to display the preprint in perpetuity. It is made available under aCC-BY 4.0 International license.

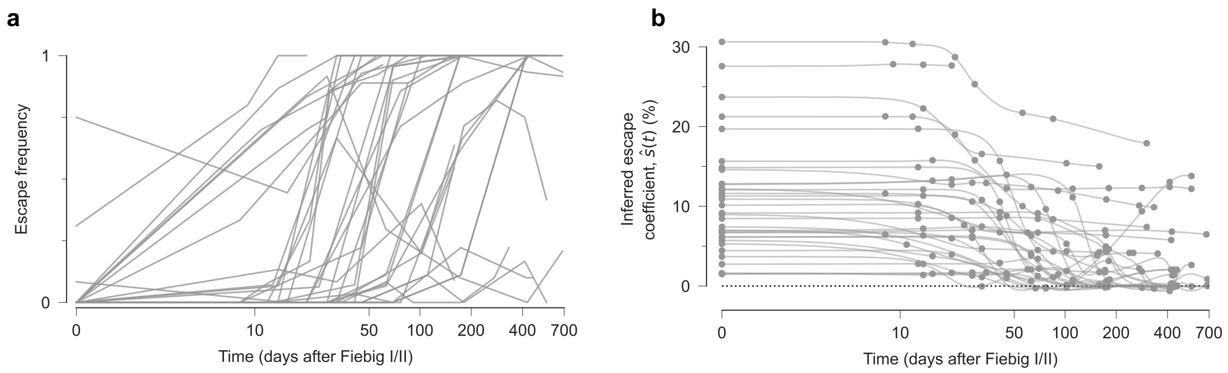

Supplementary Fig. S8. Selection for CTL escape weakens over time. **a**, Frequency trajectories for all CTL epitopes with fitness effects that could be independently estimated from data. **b**, Inferred escape coefficients over time. The pressure for immune escape is nearly always strongest during early infection but exhibits a general trend toward weaker selection over time.

bioRxiv preprint doi: https://doi.org/10.1101/2025.06.08.658500; this version posted June 9, 2025. The copyright holder for this preprint (which was not certified by peer review) is the author/funder, who has granted bioRxiv a license to display the preprint in perpetuity. It is made available under aCC-BY 4.0 International license.

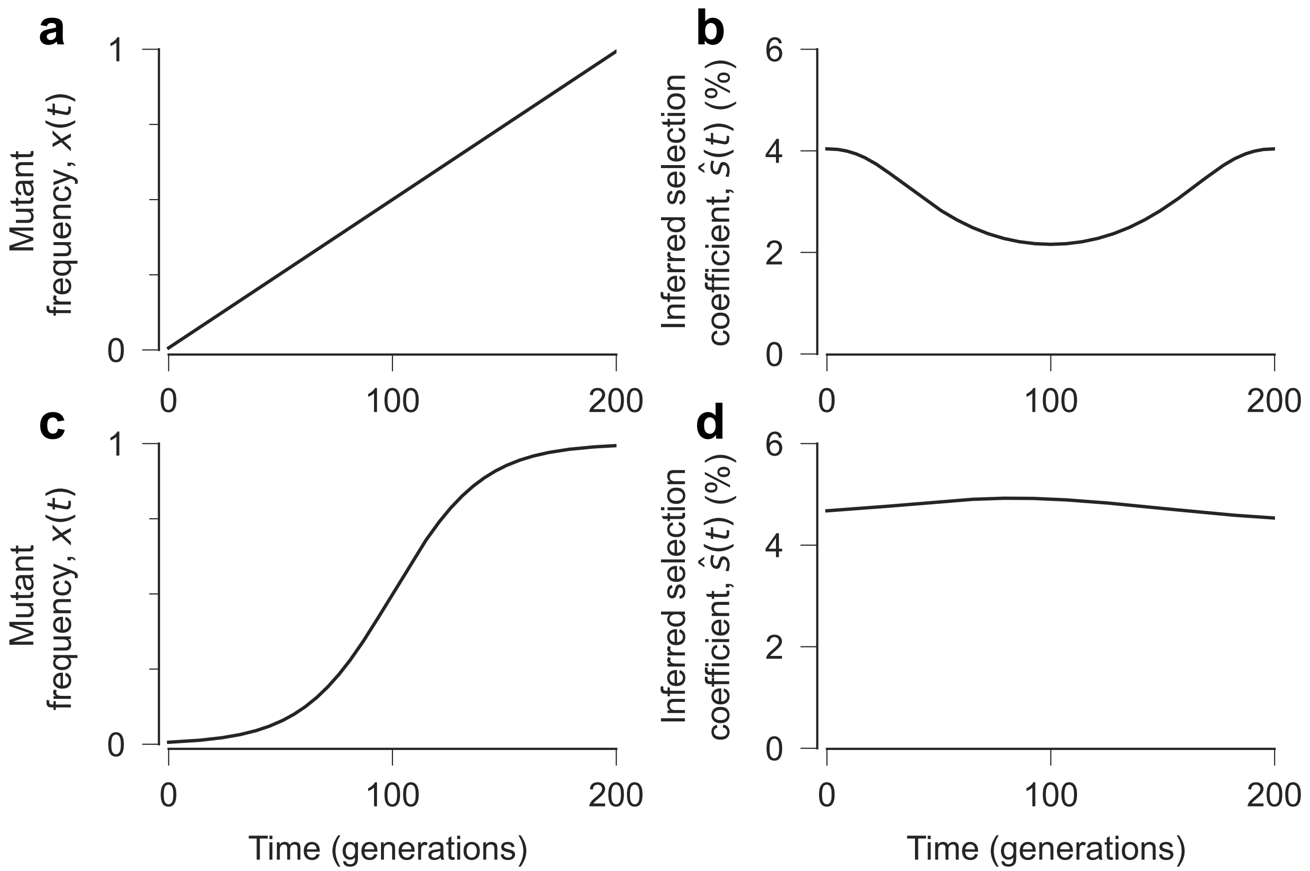

Supplementary Fig. S9. Distortion of inferred selection when allele frequencies are assumed to change by large amounts linearly in time. **a, c**, Mutant allele frequency trajectory. The frequency increases linearly in **a**, while following a sigmoid function in **c**. **b, d**, The inferred selection coefficients. Both simulations represent single allele cases without mutation or recombination. A linear increase in allele frequency does not result in a constant inferred selection coefficient; however, a sigmoid frequency trajectory yields a nearly constant inferred selection coefficient. The latter result is expected, as the evolution of a single allele frequency with a constant fitness effect should follow a sigmoidal trajectory in the absence of noise and genetic background effects. Compared to **c**, the frequency in case **a** exhibits more rapid changes in frequency during the first and last parts of the trajectory and slower ones in the middle, explaining the non-constant inferred fitness effect in **b**.
